# Bridging the missing middle in osseointegration: meso-scale topography between macro design and microroughness

**DOI:** 10.1186/s40729-025-00656-0

**Published:** 2025-11-19

**Authors:** Takahiro Ogawa, Rune Shibata, Keiji Komatsu, Takanori Matsuura, Denny Chao, Wonhee Park, Makoto Hirota

**Affiliations:** 1 Weintraub Center for Reconstructive Biotechnology, Division of Regenerative and Reconstructive Sciences, UCLA School of Dentistry, 10833 Le Conte Avenue B3-087, Box951668, Los Angeles, CA 90095-1668 USA; 2 Division of Regenerative and Reconstructive Sciences, UCLA School of Dentistry, Los Angeles, CA USA; 3 Department of Lifetime Oral Health Care Sciences, Graduate School of Medical and Dental Sciences, Institute of Science Tokyo, Tokyo, Japan; 4 Department of Periodontology, Graduate School of Medical and Dental Sciences, Institute of Science Tokyo, Tokyo, Japan; 5 Department of Dentistry, College of Medicine, Hanyang University, Seoul, Korea; 6 Department of Oral and Maxillofacial Surgery, Kyoto University Graduate School of Medicine, Kyoto, Japan

**Keywords:** Meso-scale, Osseointegration, Implant surface, Osteoblasts

## Abstract

**Purpose:**

Despite decades of clinical success with microrough implant surfaces, persistent challenges—particularly the biological trade-off between osteoblast proliferation and differentiation—highlight the need for novel surface design strategies. This review investigates the potential of meso-scale topography (10–500 μm) as a promising and underexplored dimension in implant surface engineering, situated between macro-level implant geometry and conventional microroughness.

**Methods:**

A systematic review, supplemented by a targeted literature search, was conducted to evaluate the biological and mechanical roles of meso-scale surface features on titanium, zirconia, and scaffold materials. Studies employing laser texturing, chemical etching, and 3D printing/additive manufacturing were critically assessed. Comparative insights across nano-, micro-, and meso-scale features were synthesized to delineate their distinct and synergistic contributions to osseointegration.

**Results:**

Meso-scale features confer unique biological and mechanical advantages not achievable by nano- or micro-scale designs alone. These include enhanced osteoblast recruitment/attachment, spatial organization, extracellular matrix alignment, and mechanical interlocking. Notably, meso-topography appears to resolve the classic proliferation–differentiation dichotomy observed with microrough surfaces. Many meso-scale designs also exhibit increased interfacial surface area, correlating with superior mechanical fixation. Biomimetic meso-patterns—mimicking osteoblast dimensions and native bone microarchitecture—demonstrate contact-guidance effects that promote cell alignment and matrix deposition. Most importantly, titanium and zirconia surfaces with engineered meso-topography consistently improve biological integration and biomechanical anchorage. Yet, these features remain largely absent in current clinical implants due to knowledge gaps, technical constraints, and manufacturing limitations.

**Conclusion:**

Meso-scale topography offers a powerful yet underutilized strategy to enhance osseointegration. Future implant designs should adopt an integrative, hierarchical approach that combines microroughness with meso-scale structuring to achieve synergistic improvements in cellular behavior, mechanical stability, and early healing. This strategy aligns with the hierarchical organization of natural bone and holds the potential to overcome longstanding biological bottlenecks in implant dentistry. Bridging the gap between biological potential and technological feasibility will be essential to advancing next-generation implant surface design.

**Graphical abstract:**

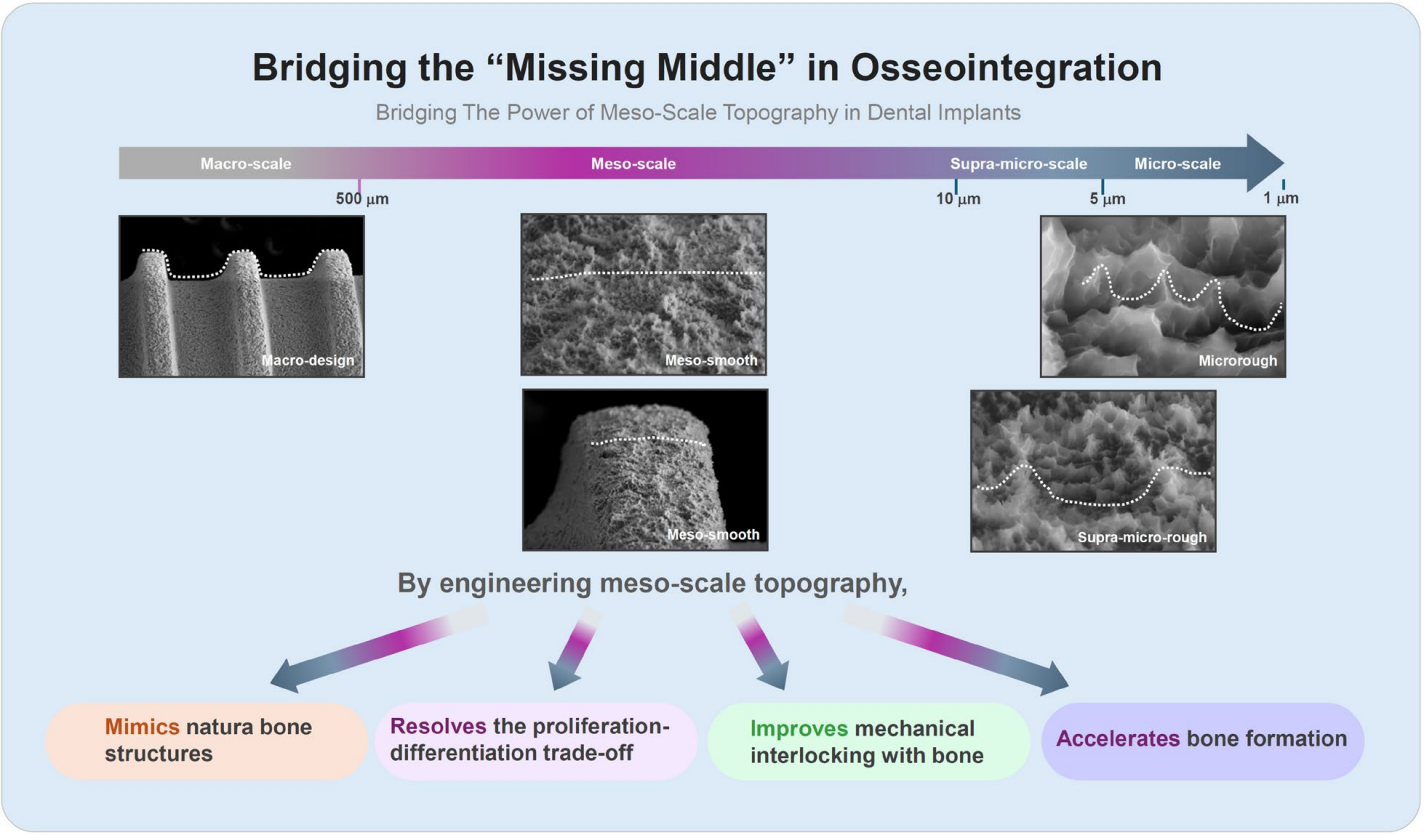

## Introduction

Surface topography at the implant–bone interface plays a pivotal role in osseointegration—the direct structural and functional connection between living bone and the surface of an implant [1–4]. Modern dental implant surfaces have historically been engineered at two distinct scales: macro-design and microroughness. At the macro-scale, parameters such as implant length and diameter, thread geometry, taper, and the presence or absence of self-tapping features are intentionally designed to engage bone mechanically, enhance primary stability, and influence load distribution [5–11]. These features are easily visible to the naked eye and are critical determinants of surgical success and short and long-term stability of implants.

**Fig. 1:**
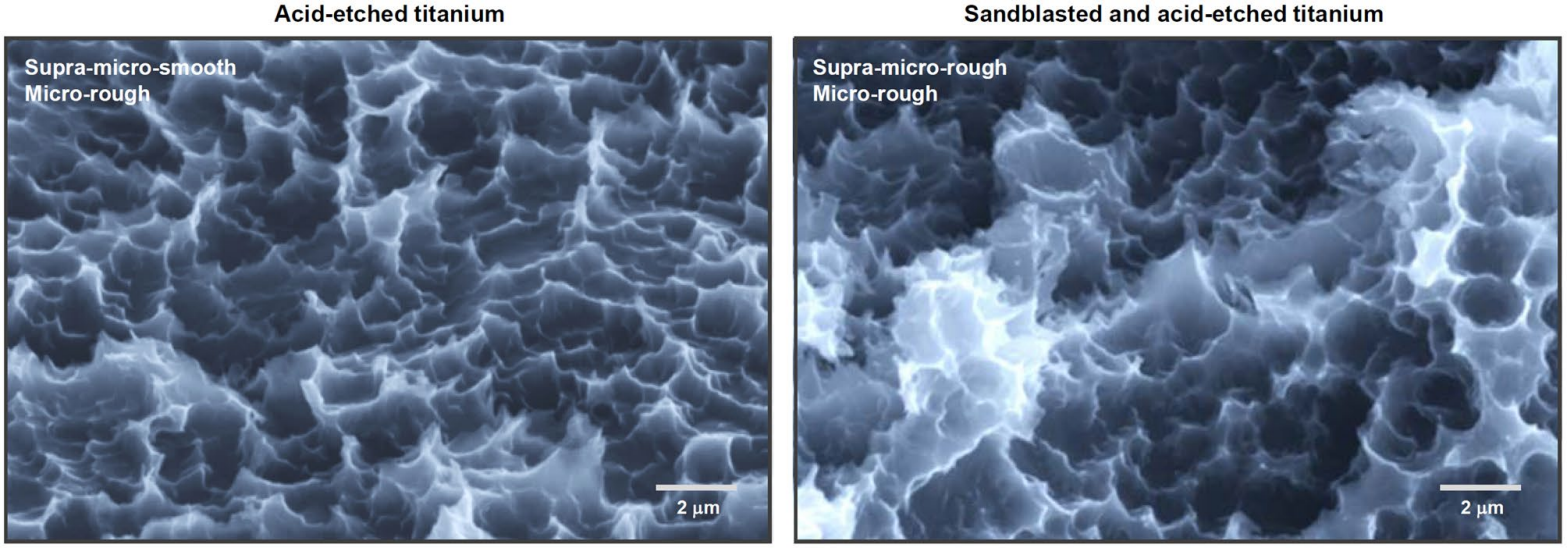
Representative microrough titanium surfaces. Scanning electron micrographs (SEM) of two widely used microrough titanium surfaces: (1) acid-etched surface and (2) sandblasted and acid-etched surface. Both surfaces exhibit irregular, isotropic microtopographies composed of micropits or microcompartments ranging from 1–5 µm in diameter—dimensions shown to favor osteoblastic differentiation. The acid-etched surface presents a relatively uniform texture, while the sandblasted and acid-etched surface introduces an additional level of complexity with supra-micron-scale features (5–10 µm) generated by pre-treatment sandblasting, creating a deep microrough surface. These topographies represent the current standard in implant surface design. However, while they enhance osteogenic differentiation, they are also associated with reduced osteoblast attachment and proliferation—a phenomenon known as the biological trade-off. This limitation underscores the rationale for exploring meso-scale surface architectures as a means to overcome micro-scale constraints and improve overall osseointegration, a central theme of this review

At the opposite end of the spectrum, micro-scale surface roughness, typically involving features in the range of 1–5 µm, has become the gold standard for surface functionalization, especially in titanium dental implants [8, 12–24]. Microrough surfaces, created commonly by acid etching or sandblasting followed by acid-etching, are characterized by a dense, isotropic distribution of micropits or microcompartments with inter-peak distances of 0.5–5 µm [19, 20, 25–32], as shown in Fig. 1. These surfaces have been shown to improve osteoblast differentiation and bone anchorage [27, 28, 33–35]. However, this approach presents a well-documented biological trade-off: while microroughness accelerates osteogenic differentiation, it often suppresses osteoblast proliferation and impairs early attachment and spreading [31, 36–44]. As a result, the actual bone-implant contact (BIC) percentage observed after sufficient healing stagnates at 45–70% in modern microrough implants [27, 45–51], well below the ideal of 100%.

In addition to these biological limitations, clinical challenges persist. These include a plateaued implant failure rate of around 8% [52–56], prolonged healing times before functional loading [57–60], and the increased risk of compromised osseointegration under systemic or local risk factors such as diabetes, osteoporosis, site development, smoking, or poor bone quality [56, 61–68]. These unsolved issues have prompted an urgent need for innovations in surface design that go beyond microroughness, yet remain distinct from macro-level design features.

Between these two well-explored domains lies a largely uncharted territory: the meso-scale. In many disciplines, “meso-scale” denotes an intermediate zone—between the small-scale molecular or cellular (micro) and the large-scale structural (macro). Applying this concept to implant science, meso-scale surface features are those that fall between the limits of micro-roughness (~ 0.5–5 µm) and macro-design (e.g., thread pitch or implant diameter). While sandblasting in implant processing does introduce some surface irregularities larger than 5 µm, the resulting features—typically 5–10 µm depressions or craters—do not clearly extend into the meso-scale regime and are categorized as supra-micron scale [35, 38].

**Fig. 2:**
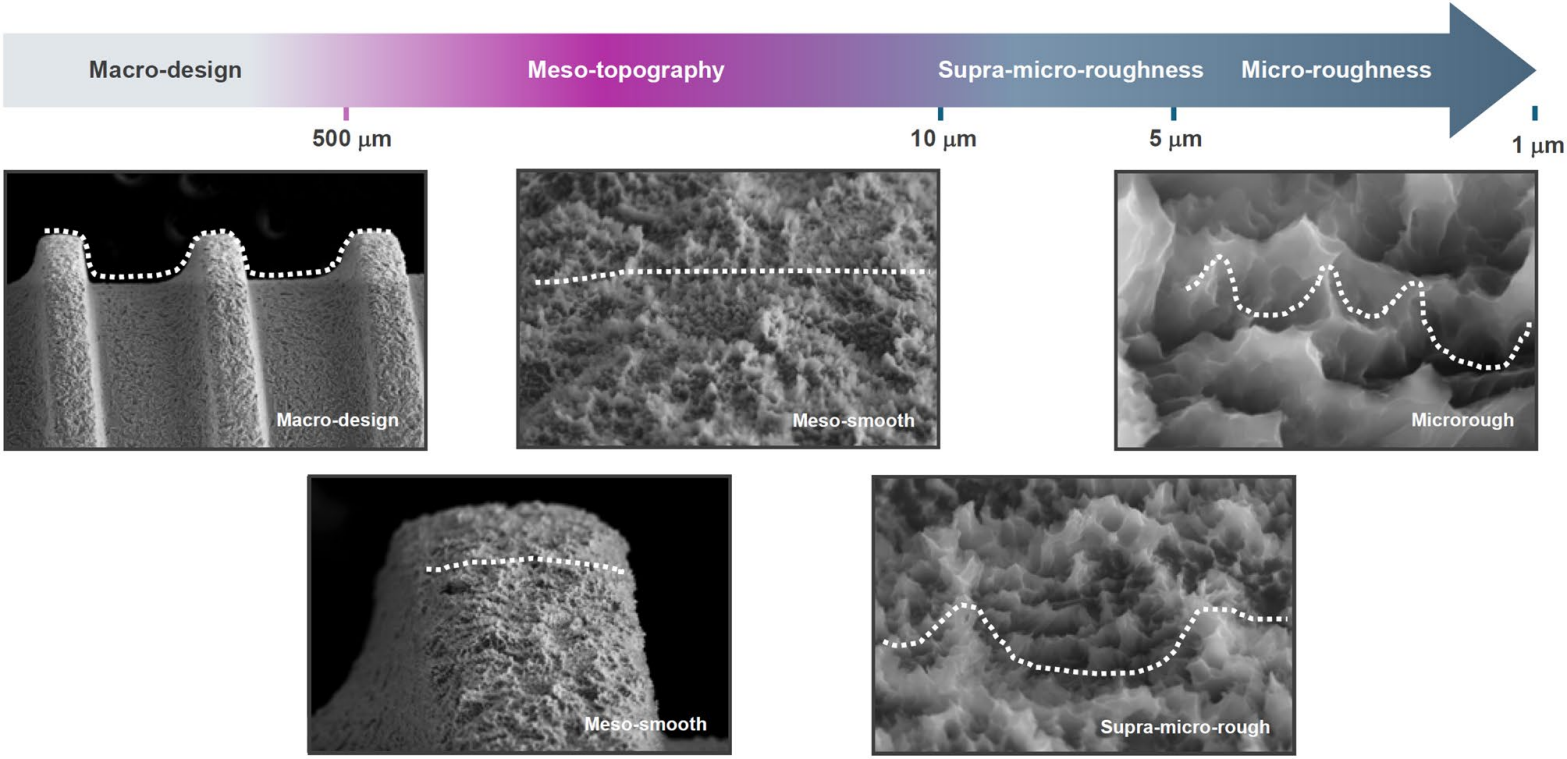
Hierarchical surface morphology of a dental implant and the missing meso-scale. Scanning electron micrographs (SEM) of a commercial dental implant are shown at progressively increasing magnifications, illustrating characteristic surface features across multiple spatial scales. A continuous scale bar denotes the hierarchy from the macro-scale (> 500 µm) down to the micro-scale (~ 1–5 µm). At the macro level, thread geometry—labeled "macro-design"—dominates and serves primarily mechanical roles, such as primary stability and load distribution. The micro-scale region, typically formed by acid etching, exhibits isotropic micropits or microcompartments that support osteoblastic differentiation and matrix production. Sandblasting introduces additional irregularities in the supra-micro range (~ 5–10 µm), adding secondary roughness atop the microrough base and creating a deep microrough surface. In contrast, the meso-scale domain (10–500 µm) appears as a design void: SEM images in this range reveal no intentional or structured topography. This lack of defined architecture is labeled “meso-smooth,” in conceptual contrast to “micro-rough” and “supra-micro-rough.” The uninterrupted, featureless appearance across the meso-range underscores a missing dimension in current implant surface design. This absent layer—positioned between macro design and microroughness—forms the conceptual foundation of this review: to investigate how meso-scale topography could serve as a critical yet overlooked contributor to improved biological integration and mechanical interlocking

**Fig. 3:**
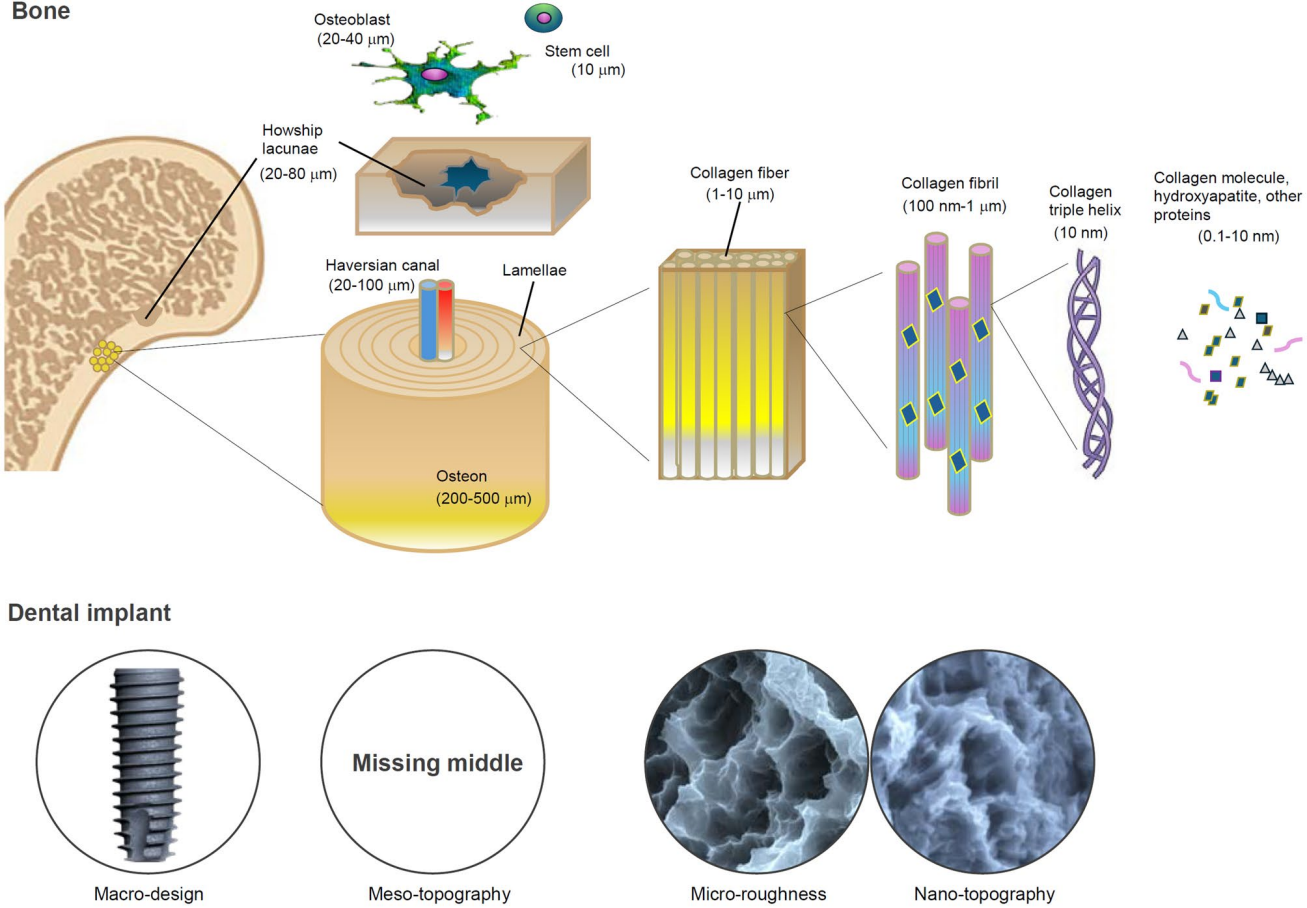
Biomimetic implants: Bridging the hierarchical gap between bone and implant surface design. This schematic compares the multiscale structural architecture of natural bone (top) with that of dental implant surfaces (bottom) across macro, meso, micro, and nano dimensions. Bone is inherently hierarchical, with specific structural units at each scale supporting distinct mechanical and biological functions: osteons and Haversian canals (meso-scale, tens to hundreds of microns) confer mechanical strength and vascular access; Howship’s lacunae and marrow niches (also meso-scale) serve as dynamic sites for remodeling and osteogenic activity; and collagen fibrils and mineral crystals at the micro- and nano-scales guide matrix organization and cellular signaling. In contrast, most current dental implants feature well-defined macro design (e.g., threads), micro-scale roughness (e.g., acid-etched micropits), and occasionally nano-scale modifications—but conspicuously lack deliberate meso-scale architecture (10–500 µm). This omission constitutes a “missing middle” in surface design, precisely within the dimensional range that aligns with key structural and biological features of bone. Embracing a biomimetic design philosophy, the incorporation of meso-scale topography presents a promising opportunity to recapitulate bone’s native hierarchy—thereby unifying mechanical interlocking, cellular accommodation, and regenerative signaling in a more physiologically relevant and integrative manner

In this review, we define meso-scale surface topography as surface features ranging from approximately 10 µm to 500 µm in size. The upper boundary is set just below the scale of implant threads—typically around 500 µm in pitch—which fall within the macro-design domain and are visible to the naked eye (Fig. 2). Meso-scale features could include well-defined spikes, grooves, ridges, concavities, pores, or pits. These structures are too large to be considered part of conventional microroughness and too small to qualify as macro-scale geometry. Occupying this intermediate range, meso-scale topographies may uniquely combine the benefits of both micro- and macro-domains—specifically, the biological modulation associated with microroughness and the physical anchoring effects attributed to macro-design. Despite their potential, current dental implant surfaces rarely incorporate such meso-scale features, as shown in Fig. 2. From a biomimetic standpoint, this missing middle is particularly significant because bone is a hierarchically structured tissue composed of features at multiple length scales—including osteons, Haversian canals, and trabeculae—many of which fall within the meso-scale range [69, 70], as illustrated in Fig. 3.

This review systematically and comprehensively synthesizes the current state of knowledge on meso-scale surface topography in the context of implant osseointegration. It combines a narrative review of well-established micro- and nano-scale surface technologies with a structured, systematic analysis of the emerging evidence surrounding meso-scale features. We summarize fabrication methods used to generate meso-scale topographies across various biomaterials—including titanium, zirconia, and scaffold-based systems—and categorize these by processing strategy (e.g., laser etching, high-temperature acid etching, additive manufacturing). The biological effects of meso-scale features are critically evaluated, with a focus on their influence on osteoblast attachment, proliferation, and differentiation in vitro, as well as bone integration outcomes in animal models. We also explore the mechanical contributions of meso-scale structures, particularly their role in enhancing implant–bone interlocking. Comparative insights between nano-, micro-, and meso-scale features are presented to delineate the distinct and synergistic roles each scale plays in osseointegration. Finally, we identify persistent gaps in current research and propose future directions to advance meso-scale surface engineering as a transformative strategy in implant design and regenerative medicine.

## Biomimetics: anatomical and physiological relevance of meso-scale morphology in bone

Bone is a hierarchically organized tissue, composed of structural elements that span several orders of magnitude (Fig. 3). At the nano-scale (1–100 nm), collagen fibrils and hydroxyapatite crystals form the fundamental building blocks of mineralized matrix [71]. At the micro-scale (~ 0.5–5 µm), the organization of osteocyte lacunae, canaliculi networks, and aligned collagen fiber bundles defines the lamellar architecture of compact bone. These micro-features guide cellular interactions, mechanotransduction, and mineral deposition. Current implant surfaces with microroughness, in a sense, accidentally mimic this scale and have demonstrated enhanced osteoblast differentiation and early-stage osseointegration [20, 72–76]. The shape of a whole bone and the thickness of cortical bone and trabecular architectures effectively formed apparently fall into macroscopic scale in bone anatomy and physiology.

**Fig. 4:**
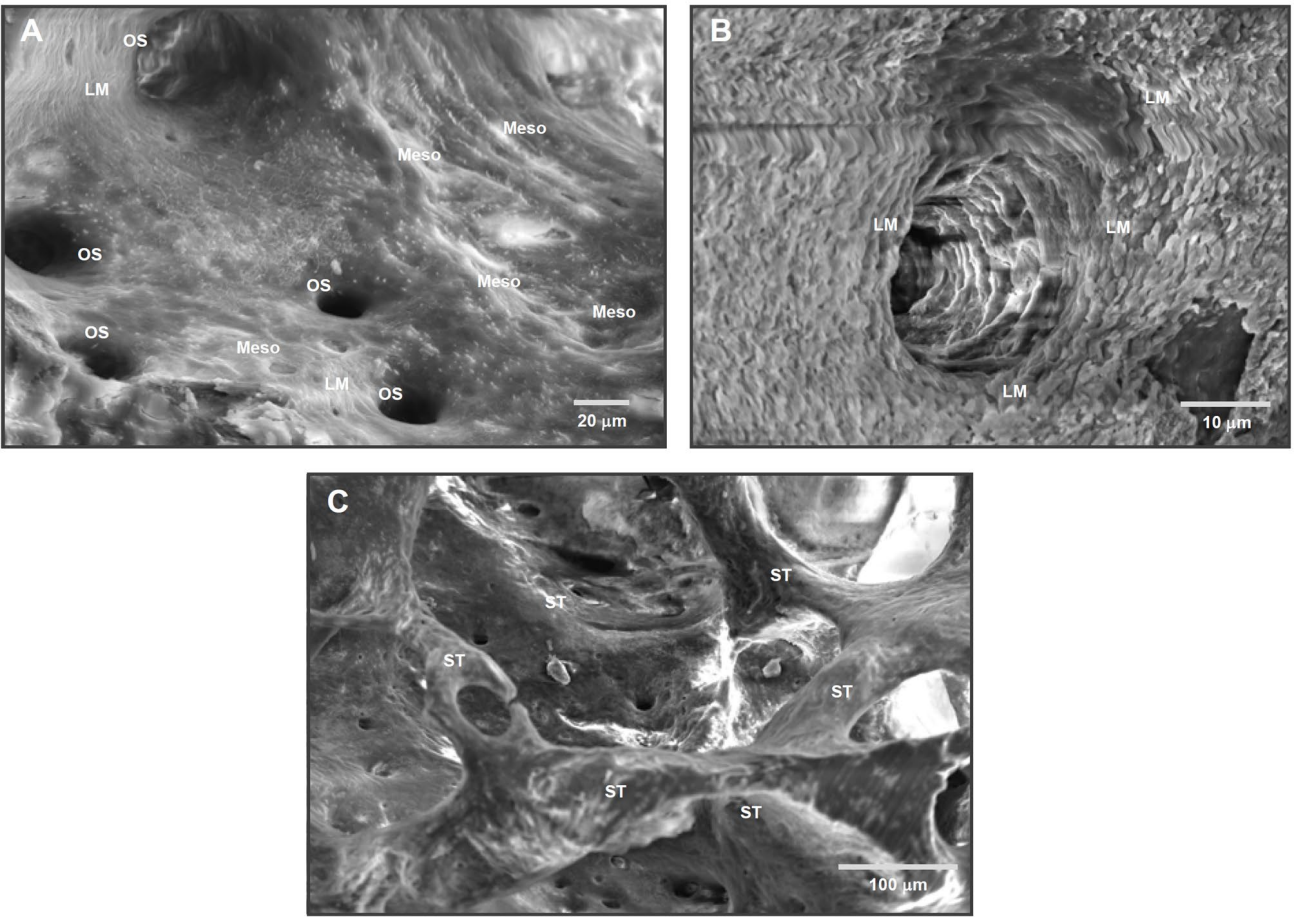
Natural meso-scale architecture of bone revealed by SEM imaging. **A** SEM images of the intaglio (internal surface) of a rat femur reveals the inherent meso-scale complexity of bone morphology. Prominent osteons (OS) with central Haversian canals—classic meso-scale structures (tens to hundreds of microns)—are surrounded by meso-scale architectural features (Meso), including ridges, depressions, lamellar folds, and marrow-facing niches. These structures collectively form a textured, cell-scaled microenvironment ideal for accommodating, guiding, and remodeling osteogenic and progenitor cells. **B** A higher-magnification view of an osteon and its Haversian canal shows the surrounding concentric lamellae (LM), emphasizing the topographically rich and organized meso-scale terrain that supports bone homeostasis and repair. **C** A Voronoi network of trabecular struts (ST) is visible within the marrow cavity, displaying branched, irregular structures that also fall within the meso-scale. These interconnected struts contribute to mechanical integrity while hosting osteogenic activity at the interface of solid and marrow spaces. Collectively, these images underscore that bone is inherently rich in meso-scale features, which are neither incidental nor decorative—but functionally essential for load distribution, vascularization, cell guidance, and regenerative dynamics. Their absence in current implant surfaces highlights a critical design gap and motivates biomimetic surface strategies

However, bridging the nano/microscopic and macroscopic domains are critical anatomical structures that reside within the meso-scale (Fig. 3). These include osteons (Haversian systems), which measure approximately 100–300 µm in diameter; Haversian canals (~ 50–100 µm); resorption pits (Howship’s lacunae); Volkmann’s canals; and trabecular bone struts, which typically range from ~ 10 to 500 µm depending on anatomical location and physiological status [70]. Representative meso-scale anatomical features within the femoral bone are shown in Fig. 4**.** These meso-scale features are essential not only for conferring mechanical strength and facilitating load distribution, but also for establishing conduits for vascular and neural networks. In addition, they serve as migration pathways for osteoblasts, osteoclasts, immune cells, and progenitors during bone remodeling and repair. The geometry and spatial distribution of these structures play a pivotal role in regulating tissue turnover and metabolic activity by facilitating the transport of signaling molecules, nutrients, and waste products across the mineralized matrix.

Indeed, during bone healing and remodeling, meso-scale structures play a central role in orchestrating the recruitment, migration, and spatial organization of regenerative cells. In this context, after an implant is placed, the formation of a stable blood clot, vascular invasion, and subsequent bone matrix deposition are highly dependent on the physical environment at this intermediate scale. Meso-scale pores and grooves in scaffolds, for instance, have been shown to promote capillary ingrowth and support tissue perfusion, accelerating healing and matrix remodeling[77, 78]. Moreover, osteoclastic resorption and subsequent osteoblastic bone formation occur along surfaces and concavities that mirror meso-scale dimensions, called Howship lacunae. Mimicking these geometric cues at the implant surface may therefore promote bone–implant integration not just via direct contact but also by creating topographies that align with the body’s native wound healing architecture[79].

Osteoblasts exhibit substantial variation in size depending on their physiological state and environment (Fig. 3). When freely circulating or in early progenitor states (e.g., mesenchymal stem cells), their diameter typically ranges from 8–12 µm [80]. Upon attachment and spreading on extracellular matrices or biomaterial surfaces, osteoblasts dramatically increase their lateral dimensions, often spreading to 20–40 µm, and in some cases beyond 50 µm, depending on substrate properties[81–85]. In confluent colonies, osteoblasts may become more compact (~ 15–25 µm), but still occupy a physical footprint within the tens of microns range. This size scale aligns with the meso-topographical window (10–500 µm), which is conspicuously absent from current implant surfaces that focus on submicron (nano) or micro-roughness (1–10 µm). Given that osteoblasts interact with the environment through spatial constraints and physical cues, meso-scale features have the potential to guide alignment, proliferation, and matrix deposition in a biomimetic manner.

Thus, the meso-scale can be understood as a critical “biological and mechanical bridge”—a transitional zone that provides spatial cues beyond the scope of molecular-scale signaling. Yet despite its evident relevance, meso-scale surface features remain largely underrepresented in contemporary implant design. This underutilization, “missing middle” represents both a conceptual blind spot and a missed opportunity in the evolution of implant surface engineering (Figs. 2 and 3). Integrating meso-scale architectures into future implant platforms could enable biomaterials to more faithfully replicate the hierarchical morphology of bone, optimize early healing dynamics, and improve osseointegration.

## Microrough surfaces: foundations, benefits, and biological trade-offs

Before delving into the emerging role of meso-scale surface topographies, it is essential to understand the well-established effects of microrough surfaces on osseointegration. Microroughness, typically defined by features in the 1–10 µm range, has been a cornerstone of dental implant surface design since the 1990s [20, 86–91]. While microrough surfaces are widely regarded as enhancing bone-to-implant contact, they also present biological limitations. This section offers a concise overview of the major biological effects of microrough surfaces, highlighting their benefits, drawbacks, and unresolved challenges to contextualize the search for next-generation implant topographies.

### Biological paradox of microroughness

#### In vitro observations: reduced attachment and proliferation

**Fig. 5:**
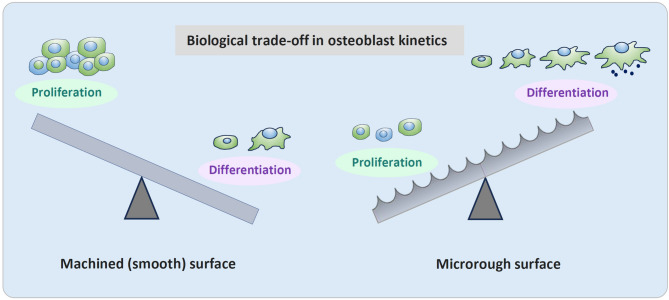
The biological trade-off in osteoblast kinetics between smooth and microrough titanium surfaces. This conceptual diagram depicts two seesaws representing the inverse relationship between osteoblast proliferation and differentiation in response to implant surface topography. On the left, a machined (smooth) titanium surface favors higher cellular proliferation but suppresses osteogenic differentiation. On the right, a microrough surface promotes osteoblast differentiation and mineralization at the expense of reduced proliferation. This biological trade-off highlights a key limitation of conventional implant surface designs—namely, the challenge of achieving both robust cell expansion and functional maturation simultaneously. The concept underscores the need for innovative surface strategies, such as meso-scale topography, to balance and overcome this dichotomy

Microrough surfaces generally show reduced osteoblast attachment and proliferation compared to machined or polished surfaces that have smoother surfaces, at the expense of promoted cell differentiation (Fig. 5). In this context, it should be noted the attachment here means that the number of cells attaching to titanium surfaces in a certain period of time, and should be clearly distinguished from the retention or adherence of cells, which is increased on microrough surfaces [92]. Although microrough surfaces enhance differentiation markers, several in vitro studies have consistently demonstrated that fewer cells attach to microrough surfaces during early culture periods [30, 31, 36]. For instance, MG63 osteoblast-like cells showed four-fold greater attachment to smooth surfaces after 24 h than rougher surfaces [93]. The same trend was confirmed on various osteogenic cell types [31, 36]. BrdU assays confirmed that proliferation on smooth surfaces consistently outpaces microrough ones, with a doubling time nearly twice as fast [30, 94, 95]. This reduced early cell density poses a limitation in generating sufficient bone-forming cells on the implant surface.

#### In vivo validation: bone-implant contact vs. bone volume

Animal models, including rabbit, pig, and rat studies, show that microrough implants consistently outperform smooth ones in mechanical removal torque and bone-implant contact (BIC) [27–29, 91, 96–99]. However, increased BIC does not always translate to greater bone volume. For example, in a rat femur model, microrough implants showed higher BIC but thinner bone layers than machined implants [27], suggesting that tighter contact, also known as contact osteogenesis, may come at the cost of volumetric bone regeneration. Indeed, the osteogenic detail profiling demonstrated that the distant osteogenesis occurring around machined smooth titanium surfaces results in higher bone volume particularly in the areas far from the surface [27].

### Functional differentiation

Microrough surfaces robustly enhance osteogenic differentiation at the cellular level, as evidenced by increased alkaline phosphatase (ALP) activity, osteogenic gene expression and protein production [43, 100–107]. Genome-wide transcriptional analysis revealed that 1.38% genes were upregulated and 0.37% were downregulated on microrough surfaces compared to machined surfaces [74]. The upregulation was also found on microrough surface in the extensive set of genes of disulfide-linked bone-related extracellular matrix (ECM) and ECM-integrin receptor interaction [74]. Targeted gene expression analyses show upregulation of Runx2, other growth factors, and signaling genes associated with cell differentiation [108–110]. However, proliferating cell nuclear antigen (PCNA) and cyclin D1, whose co-expression reduces cell proliferation, were upregulated on microrough surfaces[74]. These findings confirm a well-known biological trade-off: increased differentiation at the expense of proliferation[41–44]. Microroughness promotes earlier matrix mineralization but reduces the overall pool of proliferating osteoblasts.

### Quality of peri-implant bone, interfacial adhesion, and mechanical interlocking

Nano-indentation and scratch testing demonstrate that the bone matrix formed on microrough surfaces is significantly harder and stiffer and more adherent to the substrates than that on machined surfaces [31, 111–115]. These findings are supported by increased gene expression of proteoglycan/glycosaminoglycans and collagen synthesis [31, 33, 114, 116]. Furthermore, non-biological genuine interfacial shear strength is heavily influenced by surface area metrics on microrough titanium surfaces, such as Sdr (developed interfacial area ratio), not just by the average height (Sa) or maximum height (Sz), suggesting that topographic complexity plays a dominant mechanical role [117]. Indeed, the Sdr is nearly perfectly correlated with the interfacial interlocking between titanium and material, i.e., the larger the surface area, the stronger the retention of the material.

### Challenges and the need for surface innovation

Understanding these foundational effects allows for better identification of the existing performance ceiling—and the potential for its transcendence. Despite the benefits of microroughness, limitations such as poor initial cell attachment and reduced proliferation persist. Various surface modifications have been proposed to overcome these limitations, including UV photofunctionalization [36, 82, 94, 118–135], plasma treatment [136, 137], and hydrogen peroxide exposure [138, 139]. These techniques improve physicochemical properties of titanium surfaces. From a topographical/roughness standpoint, the rationale would be that meso-scale surface features may offer a new avenue to improve implant performance by increasing surface area and creating physical niches favorable for both cell attachment and proliferation and further enhancing mechanical interlocking.

## Nanotopography in dental implants: experimental promise vs. clinical reality

As we transition from microrough to more complex surface features, understanding the role of nanoscale topography becomes essential. While microroughness revolutionized implant integration, nanotopography was introduced to further augment biological performance. However, its translation from experimental models to clinically available implants has been inconsistent. This section outlines the biological impact, commercial efforts, and persistent limitations of nanoscale modifications.

### Experimental nanotopography: biological potential

Nanoscale features such as nodules structures have been shown in experimental models to promote osteoblast attachment, proliferation, and differentiation [40, 84, 140–142]. In these particular models, there was a clear strategy to add nano-scale features made of titanium on microrough titanium surfaces, creating a micro- and nano-hybrid rough surfaces, with a hypothesis that nano-features improve the microroughness drawback while preserving the advantages [40, 84, 143]. The hybrid micro-nano titanium surfaces were created via techniques like TiO_2_ sputter coating on pre-microroughened titanium surfaces, where nanonodules (especially around 300 nm) in a defined form were created within micropits. The surfaces significantly enhanced osteoblast behavior in vitro and increased osseointegration strength by more than three-fold compared to the microroughness alone in rat models [141]. Importantly, these hybrid surfaces seemed to resolve the classic biological trade-off of microroughness by significantly increasing the attachment and proliferation of osteoblast and not adversely affecting their differentiation [40, 141]. The effects were correlated with the size of nanonodules, providing additional evidence of direct interaction between the nanotopography and biological events [141].

### Commercial nano-featured implants

#### Inconsistent surface definition and characterization

Commercial nano-featured implants—such as SLActive®, OsseoSpeed®, and Nanotite™—claim nanoscale surface enhancements. With similar strategy to the above, attempts were made to add nano-features on the precursor microrough surfaces of the manufactures. However, SEM imaging and surface analysis reveal a lack of consistent or well-defined nanotopography. Often, nanoscale features are poorly characterized, potentially incidental, or composed of unclear chemical identification such as saline precipitates, potential fluoride composites, or calcium phosphate clusters [25, 144–152]. Increase, decrease or preservance of surface roughness parameters compared to predecessor surfaces have been contentious [37, 145, 153, 154].

#### Biological outcomes: inconsistent and limited

In vitro studies show that commercial nano-featured implants provide modest or negligible improvements in cell attachment and proliferation compared to microrough controls. In fact, when nanostructures are added to pre-existing microrough surfaces, the overall increase in surface roughness often exacerbates the drawback of microroughness—namely, reduced cell proliferation—rather than mitigating it [38, 155–157]. While some studies report elevated expression of differentiation markers such as osteocalcin and ALP [21, 93, 153, 156, 158–161], the observed effect sizes are generally small. In vivo findings mirror this trend: although early gains in BIC or removal torque have been observed on SLActive®, OsseoSpeed® surfaces, these differences tend to diminish over time, with long-term outcomes often showing no significant advantage over conventional microrough surfaces [38, 50, 146, 162]. The advantage of Nanotite surface over its microrough precursor Osseotite has been inconsistent [163, 164, 165].

### Nanotopography at a crossroads: limitations, overlaps, and the case for new directions

Experimental nano-engineered surfaces continue to show promise when built on robust microrough foundations. However, commercial applications have failed to replicate these benefits consistently, and nanotopography—at least in its current form—has not resolved the biological trade-offs introduced by microrough surfaces. Furthermore, a key complication lies in the frequent overlap between nanoscale topography and chemical modification. Most current nano-featured implants incorporate chemical alterations—such as fluoride treatment (OsseoSpeed), saline immersion (SLActive), or calcium phosphate coating (Nanotite). This overlap makes it difficult to determine whether observed biological effects arise from the physical nanotopography or from changes in surface chemistry. The resulting ambiguity compromises mechanistic clarity and hinders translational consistency. More critically, nanofeatures have not demonstrated biological advantages comparable to those achieved by microrough surfaces.

Nanotopography thus remains a technology in limbo. Moving forward, two strategic directions are evident:

1. Develop definitive nanoscale features composed exclusively of titanium, using clean and controlled fabrication methods that eliminate unintended chemical artifacts.
2. Shift toward meso-scale surface engineering, which offers a new design dimension aligned with native bone architecture and has shown superior influence on cell organization, vascularization, and mechanical integration—as will be discussed in subsequent sections.

## Supra-micron roughness: an auxiliary enhancer, not a trade-off solution

As introduced earlier, supra-micron roughness (typically 5–10 µm in feature size) is commonly implemented as a pre-treatment step—most notably through sandblasting—prior to acid etching in the fabrication of dental implants. This treatment produces larger-scale irregularities, depressions, dents, or craters that are superimposed onto the finer microroughness created by acid etching. Although these features do not constitute a distinct or novel scale of roughness, they play an auxiliary role in enhancing surface area and mechanical interlocking by creating a deep microrough surface. Traditional sandblasting uses 100–250 µm Al₂O₃ particles to create micro-scale irregularities (~ 1–2.5 µm Sa). However, employing coarser particles (250–500 µm) can generate larger irregularities, albeit in a less orderly and less uniform fashion than etching-based techniques.

Mechanical testing between titanium and polymethyl methacrylate (PMMA) bone cement has shown that sandblasted titanium surfaces exhibit 3.5-fold higher shear strength than machined surfaces, with Sa values increasing fourfold [117]. Moreover, when combined with acid etching, sandblasting improved the shear strength by 1.5-fold relative to acid etching alone. Although some roughness gains are partially lost during the etching step, the mechanical advantage remains significant.

In vivo studies confirm this synergy: implants with sandblasted and acid-etched surfaces demonstrate higher removal torque values than acid-etched-only implants, indicating superior mechanical interlock and osseointegration [166]. From a cellular perspective, sandblasted surfaces promote osteogenic differentiation. One study reported significantly elevated osteocalcin expression on sandblasted titanium compared to smooth controls [101]. Interestingly, alkaline phosphatase activity—a marker of early differentiation—did not significantly differ among surfaces treated with various TiO₂ grit sizes (63–90, 106–180, 180–300 µm), suggesting that the differentiation advantage may be offset by the reduced cell proliferation.

Another study examining sandblasted surfaces with Ra values of 2.0, 3.0, and 3.3 µm demonstrated a clear biological trade-off: as surface roughness increased, osteoblast proliferation declined, while osteocalcin expression—a marker of differentiation—was upregulated [102]. Mechanical testing revealed that the rougher surfaces required more than twice the torque for removal compared to smooth implants, confirming significantly enhanced mechanical interlocking. Similarly, another study reported that fewer osteoblasts were initially recruited to sandblasted surfaces than to machined ones; however, once attached, these cells exhibited greater resistance to dislodgement under mechanical stress [92].

**Fig. 6:**
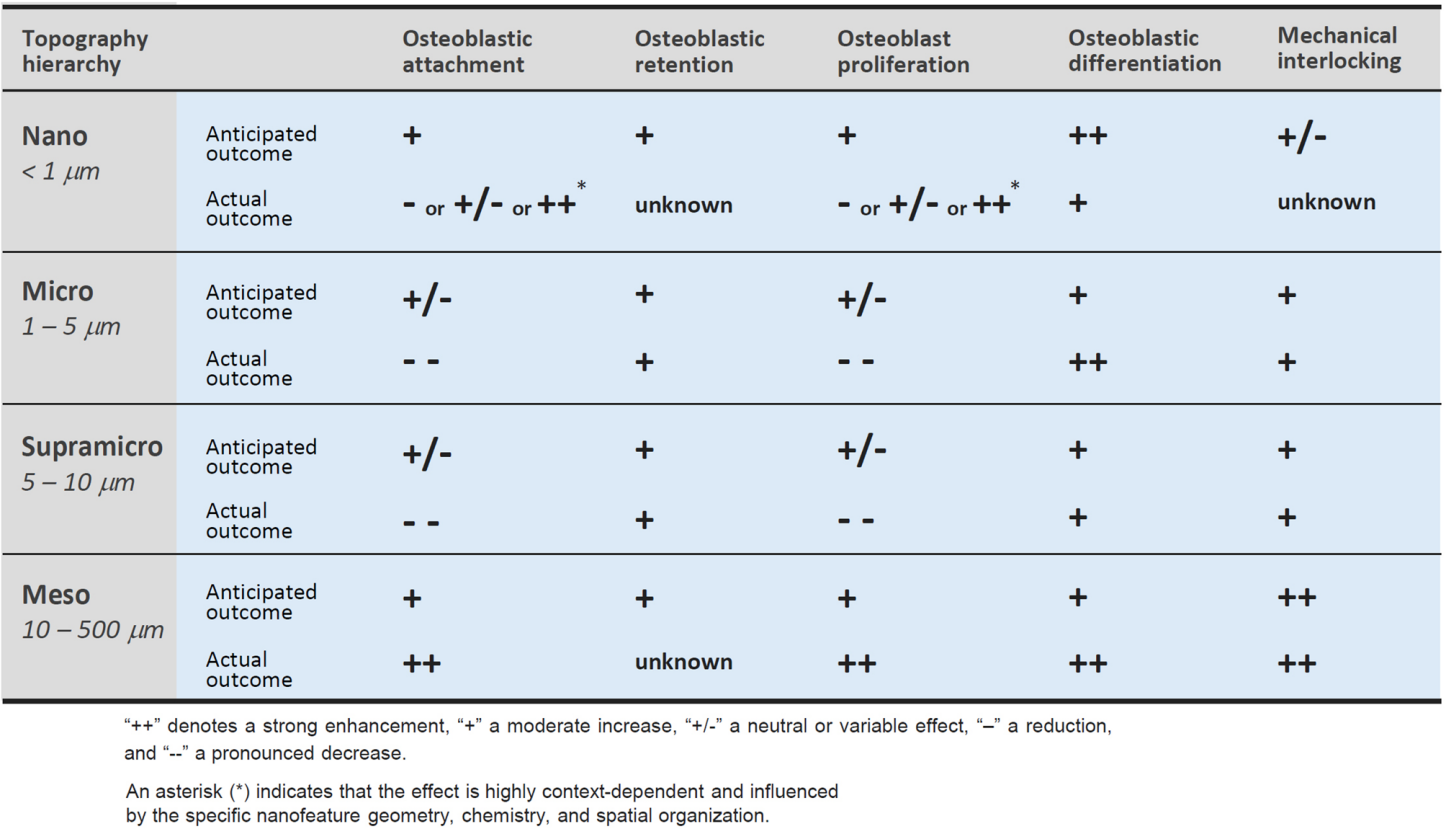
Hierarchical effects of surface topography scale on osteoblast behavior and mechanical interlocking. This comparative diagram summarizes how surface features at different hierarchical scales—nano, micro, supra-micro, and meso—affect key biological and mechanical responses relevant to osseointegration. Each topography scale is assessed both by anticipated effects based on theoretical expectations (e.g., increased surface area, cell-scale geometry), and by actual effects as reported in the literature. Notably, meso-scale topography shows consistently positive or synergistic effects across all categories, suggesting its unique and underutilized value in future implant surface design

In summary, supra-micron roughness enhances mechanical interlocking and promotes osteogenic differentiation. However, it also reduces osteoblast recruitment and proliferation, reinforcing the classical biological trade-off rather than resolving it. Therefore, while supra-micron features serve as an auxiliary enhancer to microrough surfaces, they do not represent a distinct topographic domain nor provide a solution to the limitations of microroughness. Their value lies mainly in mechanical augmentation—not in overcoming the biological constraints. Future surface designs must look beyond this scale to achieve true biological and mechanical harmony. The role of nano-, micro-, and supra-micro-features in biological events of osteoblasts are summarized in Fig. 6. Note that the biological outcomes actually proven were often opposite to the anticipated effects.

## Meso-scale surface engineering: bridging macro and micro

**Fig. 7:**
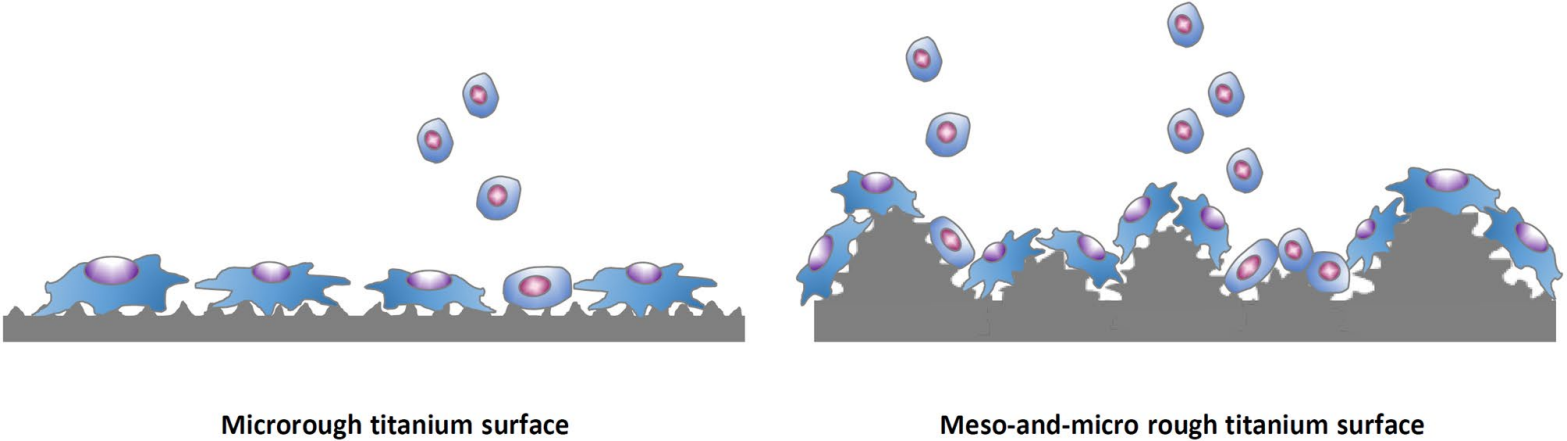
Strategic integration of meso-scale topography with microroughness to mitigate the biological trade-off in osteoblast kinetics. This schematic illustrates a conceptual comparison between a conventional microrough implant surface and a hybrid surface that incorporates superimposed meso-scale spikes onto a microrough background. While microrough surfaces are known to promote osteoblast differentiation, they tend to suppress proliferation—a well-recognized biological trade-off. The hybrid surface introduces meso-scale architectural features to increase three-dimensional spatial complexity and surface area, thereby providing additional niches for cell attachment and expansion. This design strategy aims to accommodate more osteoblasts during the early healing phase

The meso-scale domain (10–500 µm) represents a critical yet underexplored dimensional range in implant surface engineering. While micro- and nano-scale modifications have dominated the landscape of dental implants over the past decades, emerging evidence suggests that meso-scale features—such as ridges, spikes, grooves, and pores—may offer unique biological advantages. These include enhanced guidance of multicellular behavior, improved mechanical interlock with bone, and facilitation of vascular infiltration. Importantly, the meso-scale range encompasses the dimensions of key anatomical and structural elements of bone, including osteons, Howship lacunae, Haversian canals, and trabeculae (see Sect. "Biomimetics: Anatomical and physiological relevance of meso-scale morphology in bone". Biomimetics), offering a geometrically relevant platform for tissue-level interaction and remodeling. As such, integrating meso-scale design may serve not only as a complementary strategy but as a new paradigm that bridges macro-mechanical design with cell-level biological function. Technically and specifically, a strategy would be to provide more space for cellular settlement and more geometrical cues for the cells (Fig. 7).

Unlike micro- or nano-scale features, which can often be generated through established methods such as acid etching and sandblasting, meso-scale features require more targeted, geometry-specific fabrication techniques. These include laser ablation, additive manufacturing, and high-temperature acid etching, among others—often customized for the base material. This section categorizes current meso-scale fabrication strategies by material class—titanium, its alloys, zirconia, and scaffolds—and examines the morphological outcomes and biological responses associated with each.

### Search strategy and article selection

A targeted literature search was conducted using the PubMed database, following a structured keyword-based approach to identify peer-reviewed studies related to meso-scale surface engineering in biomaterials. The following Boolean search strings were applied individually and in combination: “meso-scale AND titanium,” “meso-scale AND alloy,” “meso-scale AND scaffold,” “meso-scale AND zirconia,” and “meso-scale AND biomaterial.” Only articles published in English and involving applications in bone biology (osteoblasts, osteogenesis, osseointegration) and biomedical applications were included. Studies involving non-biological contexts (e.g., industrial or fluidic systems), those without surface characterization at the meso-scale, or studies using “meso-scale” in unrelated contexts (e.g., computational modeling, bulk material analysis, or biological tissue) were excluded.

**Fig. 8:**
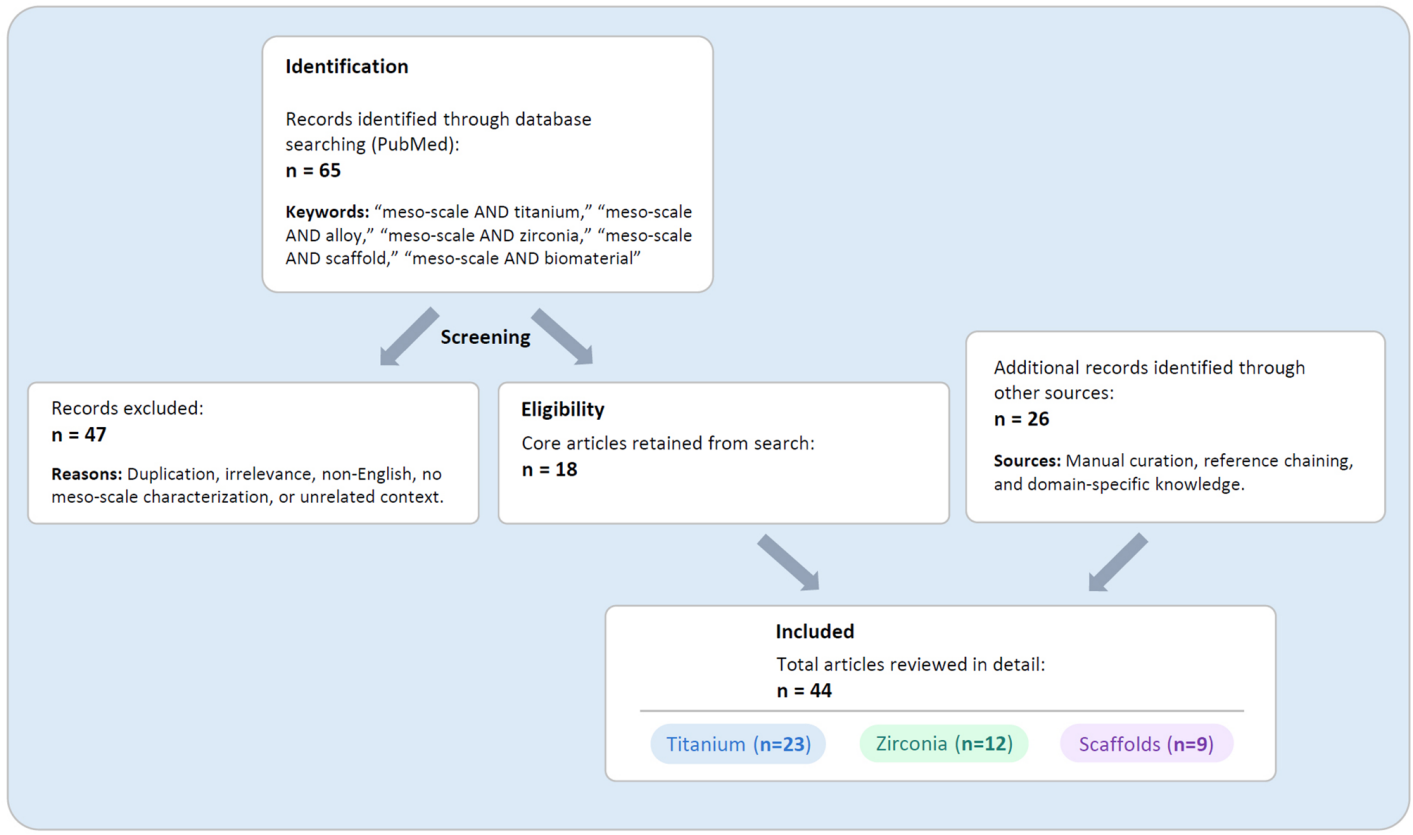
Flowchart illustrating the study selection process for the systematic review on meso-scale surface texturing/engineering in biomaterials. A structured PubMed search was conducted using defined Boolean strings targeting meso-scale features on titanium, zirconia, and scaffold materials relevant to osseointegration

After initial screening, 65 articles were retrieved. Of these, 47 were excluded based on duplication, irrelevance, or failure to meet inclusion criteria. The remaining 18 articles were included as the core dataset. An additional 26 relevant studies were identified through manual curation, reference chaining, and domain-specific knowledge. This yielded a total of 44 articles reviewed in detail in this section, comprising 23 studies on titanium, 12 on zirconia, and 9 on scaffold-based systems. The selection process is illustrated in Fig. 8. A detailed summary of the reviewed studies is organized into four tables according to material type and fabrication method: Table 1 (titanium surfaces modified by acid etching and laser etching), Table 2 (titanium surfaces fabricated via 3D printing or additive manufacturing), Table 3 (zirconia-based surfaces), and Table 4 (scaffold structures with meso-scale architecture). Clinical studies and review articles were excluded from the summary tables to ensure consistency in data interpretation and focus on primary experimental evidence.

**Table 1:** Summary of reviewed studies on meso-scale titanium surfaces created by chemical and laser etching

References	Author (year)	Method for meso-scale creation & material type	Surface morphology & topography	Biological outcomes
*Chemical etching*				
[167]	Hasegawa (2025)	Method: High-temperature acid-etching (sulfuring acid) Material: cpTi (grade 2)	A unique hierarchical surface with meso-scale spikes (10–80 µm wide), micro-scale compartments, and nano-scale polymorphic structures. The size and density of meso-spikes increased with etching temperature	The hierarchical surface significantly promoted osteoblast differentiation (ALP, Opn, Ocn gene expression) and in vivo osseointegration strength without compromising initial cell attachment. Osseointegration was highly correlated with the size and density of the meso-spikes
*Laser etching*				
[175]	Chen (2024)	Method: Laser irradiation (pulsed Nd:YVO_4 laser) Material: ASTM Grade 4 Ti	Created a microchannel structure with an average width of 21 µm and depth of 18 µm. The surface also had a complex nanoscale structure on top of the microchannels	The laser-produced microchannels enhanced pre-osteoblast proliferation, upregulated the expression and secretion of osteogenic differentiation markers (eg., COL1A1, SPP1), and increased extracellular mineralization compared to ground or SLA surfaces
[176]	Maalouf (2022)	Method: Femtosecond laser (FSL) texturing using linear vs. azimuthal polarization Material: Ti-6Al-4V	Created Laser-Induced Periodic Surface Structures (LIPSS), long (> 10 m) streaks with nanoscale roughness. Linear polarization created an anisotropic surface, while azimuthal polarization created an isotropic (radial) surface	Isotropic (radial) LIPSS surfaces enhanced osteoblastic differentiation by increasing cell contractility, fibronectin production, osteogenic gene expression (ALP, osterix), and final mineralization compared to anisotropic or polished surfaces
[177]	Zwahr (2017)	Method: Direct Laser Interference Patterning (DLIP) Material: cpTi (grade 4)	Created periodic line-like patterns with meso-scale spatial periods of 5, 10, and 20 µm and structure heights Sz = 2.4 μm (Sa = 0.15 μm). The treatment also increased nitrogen content in the surface reactive layer	Human osteoblast viability was 16% higher on the 20 µm patterned surface compared to grit-blasted/acid-etched controls. This was attributed to enhanced cell metabolism, not increased cell number. Cells showed contact guidance along the lines
[178]	Chao (2024)	Method: Laser texturing (Laser-Lok) on healing abutments Material: Titanium	A hybrid topography with monodirectional meso-scale channels (15-µm pitch) and woven oblique micro-ridges within the channels	While fibroblast attachment and proliferation were unchanged, cells on the laser-textured surface aligned with the mesochannels (contact guidance) and were 1.8 times more resistant to detachment. Adhesion-related genes (fibronectin, integrin β-1) were upregulated
[179]	Lee (2015)	Method: Femtosecond laser dimpling Material: cpTi (grade 4)	Created micro-dimples with a 5 µm diameter on a polished Ti surface, with center-to-center spacing of either 15 µm or 30 µm	Epithelial-like cell adhesion strength was significantly higher on the dimpled surfaces compared to the polished control. Fibroblast adhesion was not significantly affected. Both cell types showed increased expression of adhesion proteins around the dimples
[180]	Tiainen (2019)	Method: Laser Surface Texturing (LST) with a Q-switched Nd:YAG laser Material: Ti-6Al-4V	Created nine different cross-hatched micropatterns with groove widths of 40, 80, and 140 µm and spacings of 0, 20, and 100 µm	This study focused on mechanical properties, not direct cell response. All patterned surfaces had a higher coefficient of friction against bone compared to grit-blasted controls, suggesting improved primary stability
[182]	Gotz (2004)	Method: Laser texturing (Nd:YAG) to create pores, with some surfaces also corundum-blasted Material: Ti6Al4V	Defined meso-scale pore sizes of 100, 200, and 300 µm were created. Blasting created a rough (Ra = 7.25 µm) inter-pore surface	Combining laser-texturing with surface blasting profoundly improved osseointegration. Bone remodeling occurred in all pore sizes, though with a time lag in 100 µm pores. 200 µm was suggested as the optimal pore size
[183]	Xue (2007)	Method: Laser Engineered Net Shaping (LENS™) to create porous structures by partial melting Material: cpTi	Porous structures with controlled porosity (17–58%) and pore sizes up to 800 µm. Surfaces were naturally rough, composed of partially melted spherical particles	Porous Ti surfaces enhanced cell proliferation and stimulated faster osteoblast differentiation (higher ALP and ECM) compared to polished Ti. A critical pore size of > 200 µm was identified as necessary for cells to grow into the pores. In smaller pores (< 150 µm), cells bridged over the surface instead of penetrating

**Table 2:** Summary of reviewed studies on meso-scale titanium surfaces fabricated by 3D printing/additive manufacturing

References	Author (year)	Method for meso-scale creation & material type	Surface morphology & topography	Biological outcomes
[188]	Shu (2025)	Method: Selective Laser Melting (SLM) vs. Sandblasted/Acid-Etched (CNC-SLA) Material: Ti-6Al-4V ELI	SLM implants had a significantly rougher (Sa = 13.45 µm) and more rugged surface composed of partially melted spherical particles compared to the moderately rough (Sa = 1.57 µm) CNC-SLA surface	Histological osseointegration (BIC, BV/TV) was similar between groups, but SLM implants showed significant microscale bone interlocking after week 2. Removal torque strength was significantly higher for SLM implants by week 2. Microscale bone interlocking on the rough SLM surface is a key biomechanical mechanism that alleviates peri-implant bone strain and enhances osseointegration strength
[189]	Sun (2022)	Method: SLM combined with sol–gel coating of Mesoporous Bioactive Glass (MBG) nanospheres Material: cpTi	A unique dual micro/nano-topography was created by coating the primitive meso-sized (20–40 µm) SLM particles with nano-sized (230–300 nm) MBG spheres. The MBG coating turned the hydrophobic SLM surface superhydrophilic	The MBG-SLM-Ti surface showed enhanced osteoblast adhesion and significantly higher osteogenic differentiation (ALP activity) compared to MBG on a smooth surface. This novel approach preserves the native SLM surface roughness, using the micro-particles as a scaffold for a bioactive nano-coating, thus enhancing bioactivity without needing complex post-processing to remove particles
[190]	Abar (2021)	Method: Laser Powder Bed Fusion (L-PBF) with post-processing (Polished, Blasted) or with artificially printed topography (Sprouts) Material: Ti6Al4V ELI	Samples ranged from smooth (Polished, Ra = 0.5 µm) to very rough (Rough Sprouts, Ra = 9.9 µm). The printed features were on the mesoscale (500 µm height)	Artificially printing large, rough features diminished mechanical properties (tensile strength, ductility). There was no clear benefit to cell proliferation or differentiation; in fact, the roughest groups showed decreased expression of the osteogenic marker Bglap. Artificially printing mesoscale roughness is not an effective strategy to promote osseointegration, likely because the features are too large to influence cellular-level activity
[191]	Peng (2016)	Method: SLM-fabricated multi-rooted implant (MRI) with a designed porous structure vs. commercial RBM-treated implant Material: Ti6Al4V	The SLM implant had a designed, interconnected porous surface with pore sizes of 300 µm in the cortical region and 400 µm in the cancellous region	Bone grew into the MRI's pore structures after 4 weeks. The MRI group showed higher bone volume density and significantly stronger biomechanical fixation (push-out and torque tests) than the control. SLM enables the fabrication of complex, multi-rooted implants with designed porosity that mimics bone structure, leading to improved bone ingrowth and mechanical interlocking
[193]	Jin (2022)	Method: Direct Metal Laser Sintering (DMLS) and Electron Beam Melting (EBM) with small (22.5 µm) and large (100 µm) particles; some samples were acid-etched Material: Ti6Al4V	EBM surfaces were smooth and stripy with few particles. DMLS surfaces were rough with many partially sintered particles. Acid etching removed small particles but not large ones	Surfaces without particles (EBM and acid-etched DMLS with small particles) favored osteogenic differentiation. Un-printed small particles stimulated the highest pro-inflammatory cytokine expression. The choice of 3D printing method and post-processing should depend on the clinical application. Surfaces with particles may be prone to wear debris and inflammation, suggesting smooth or particle-free surfaces are better for joint prostheses
[194]	Shahsavari (2022)	Method: Electron Beam Manufacturing (EBM) with different surface finishes: as-printed (AP), mechanically polished (MP), and Electron Beam Surface Remelting (EBSR) Material: Ti-6Al-4V	EBM-AP surface was very rough (Ra = 24.83 µm). The novel EBSR technique effectively smoothed the surface by re-melting the top layer, reducing roughness by 82% to Ra = 4.55 µm	No biological outcome was reported. This study focused on corrosion. The smoother surfaces created by EBSR and MP showed significantly enhanced corrosion resistance. Surface roughness was a more dominant factor for corrosion behavior than the underlying material microstructure. Electron Beam Surface Remelting (EBSR) is a promising in-situ or ex-situ technique for improving the surface finish and corrosion resistance of AM titanium parts without mechanical contact or chemical agents
[195]	Shaoki (2016)	Method: SLM vs. machined (MA) and commercial Nobel-speedy implants Material: cpTi	SLM implants had a porous and highly rough surface (Ra = 10.65 µm) with partially melted particles	In vitro, the rough SLM surface enhanced osteoblast proliferation and ALP activity compared to the smooth MA surface. In vivo, osseointegration (BIC, BV/TV) was comparable among all groups, but the removal torque of SLM was lower than the commercial implant. Although promising in vitro, the native, highly rough SLM surface did not outperform a commercial implant in vivo. SLM technique holds promise and additional surface modifications may be beneficial on SLM surfaces

**Table 3:** Summary of reviewed studies on meso-scale zirconia surfaces

References	Author (year)	Method for meso-scale creation	Surface morphology & topography	Biological outcomes
*Laser Ablation / Etching*				
[196]	da Cruz (2022)	Nd:YAG Laser Machining	Microgrooves of varying dimensions were added to sandblasted/acid-etched surfaces (~ 45 µm width/50 µm depth to ~ 125 µm width/23 µm depth)	No Significant Effect: Adding laser grooves did not significantly alter cell viability, proliferation, or differentiation compared to the control surface. The control was a clinically relevant sandblasted and acid-etched surface, suggesting these specific laser grooves offered no additional benefit to an already micro-roughened surface
[197]	Rezaei (2018)	Solid-State Laser Sculpting	Hierarchical surface with meso-scale grooves (50 µm wide, 6–8 µm deep), micro-scale valleys, and nano-scale nodules	Accelerated Osteoblast Differentiation: Gene expression was 7–25 times higher on the hierarchical surface. Enhanced Osseointegration: Strength of bone integration was doubled compared to the machined control. Proliferation was not compromised. The hierarchical structure promoted differentiation without the negative effect on proliferation often seen with rough titanium
[200]	Baino (2019)	Pulsed Nd:YVO₄ Laser Radiation	Created various textures, including a pattern of square "hills" and valleys. Surface roughness (Rₐ) was modulated from 3 to 30 µm	No biological data. The study concluded that the created roughness (15–30 µm) was on the same scale as osteoblasts and was therefore expected to promote bone interlocking. A materials science study focused on texturing alumina/zirconia composites for hip joint prostheses
[201]	Saruta (2021)	Solid-State Laser Etching (Crisscrossing)	Biomimetic design featuring meso-scale cactus-inspired spikes (60 µm wide; 20–80 µm height) combined with nano-scale trabeculae	Optimized Meso-Structure: Osteoblast differentiation and in vivo bone integration peaked on surfaces with 40 µm-high spikes. Strength was superior to acid-etched titanium. Focused on optimizing the meso-scale spike height for maximum biological response. A prototype implant with the optimized texture was created
[202]	Kitajima (2022)	Laser Etching	Biomimetic design featuring meso-scale cactus-inspired spikes combined with nano-scale trabeculae. Fixed meso-scale spikes (40 µm height) with two different nano-trabecula sizes (small: ~ 134 nm, large: ~ 237 nm)	Minor Nano-Scale Effect: Small nano-trabeculae slightly enhanced osteoblast differentiation, but the effect was minimal compared to the overall benefit of the meso-nano hybrid topography. Concluded the meso-structure is the dominant factor for biological response in this model, not the nano-structure variance
[203]	Taniguchi (2015)	Fiber Laser Irradiation	Regular grooves and fine crevices creating a rough surface. Average roughness (Sₐ) increased from 0.18 µm to 1.75 µm	Improved Osteogenesis: Cell proliferation, ALP activity, and calcification were all significantly greater on the rough surface. Enhanced Osseointegration: In vivo, the rough surface showed a higher BIC ratio and removal torque. A foundational study demonstrating that fiber laser-induced roughness on Y-TZP supports osseointegration. Control was machine-surfaced zirconia
[204]	Kitajima (2023)	Computational Fluid Dynamics (CFD) Modeling	Modeled surfaces including a hybrid meso-nano (40 µm spikes + 300 nm nodules)	Superior Blood/Protein Recruitment: The hybrid surface was most effective at recruiting blood and fibrinogen to the implant interface and significantly slowed blood velocity, creating a favorable microenvironment for protein and cell attachment. A computational study providing a mechanistic explanation for the superior biological performance of the hybrid meso-nano topography
*Additive Manufacturing (3D Printing)*				
[205]	Zhang (2022)	Additive Manufacturing (Nano-particle ink-jetting)	A dense implant core seamlessly integrated with a porous surface layer containing directional, lamellar pores (~ 37 µm deep, ~ 5 µm wide)	Favorable Cell Response & High Strength: The directional pores guided osteoblasts into an elongated, uniformly oriented morphology. The implant showed high mechanical strength, which increased after aging. A novel method to create integrated surface porosity during manufacturing, eliminating post-processing and the risk of coating delamination
[206]	Nakai (2022)	Additive Manufacturing (Stereolithography)	As-sintered surface with inherent micro-scale roughness from the AM process (Sₐ ~ 0.28 µm). No specific meso-structure was created	Comparable to Titanium: Cell viability, ALP activity, and Type I collagen gene expression on AM zirconia were comparable to or better than those on the SLA-titanium control. Showed that even without specific texturing, the as-sintered AM zirconia surface provides a strong biological response, outperforming AM ATZ

**Table 4:** Summary of reviewed studies on meso-scale scaffold structures

References	Author (year)	Method for meso-scale creation & material type	Surface morphology & topography	Biological outcomes
[209]	Krieghoff (2019)	Method: Templating with solid lipid particles. Material: Lactic acid-based macromer (TriLA)	Created macroporous scaffolds with a continuous pore network. Optimized fabrication ("HiPo") increased the mean pore size to ~ 210 µm with a broad distribution up to 400 µm	Improved Coating and Mineralization: Scaffolds with larger pores (HiPo) allowed for more efficient coating with collagen and sulfated hyaluronan (sHA3), which in turn significantly enhanced osteoblast ALP activity and bone matrix formation. Increasing the meso-scale pore size of a synthetic polymer scaffold can improve its capacity for bio-functionalization and subsequent osteogenic potential
[210]	Gilchrist (2014)	Method: Micro-photopatterning (µPP). Material: Fibronectin on a non-fouling hydrogel substrate	Investigated the interaction between micro-scale cues (~ 2 µm lines) and meso-scale boundary confinements (> 100 µm), such as elongated rectangles (1250 µm × 125 µm)	Meso-Scale Cues Dominate Alignment: While micro-patterns guided single cells, high aspect ratio meso-scale boundaries dominated the alignment of multicellular tissues, leading to highly organized and increased fibrillar collagen deposition. A critical meso-width of ~ 500 µm was identified. A fundamental study demonstrating that meso-scale geometric confinement can override micro-scale topographical cues to direct collective cell behavior and tissue organization
[211]	Laubach (2023)	Method: 3D Printing (Additive Manufacturing). Material: Polycaprolactone-Hydroxyapatite (mPCL-HA) composite	Scaffolds with a Voronoi tessellation design, creating a biomimetic, trabecular-like structure with irregular polyhedral pores and high interconnectivity (~ 73% porosity). Filament diameter was ~ 120–155 µm	Excellent Biocompatibility and Bone Formation: Scaffolds showed slow degradation and, when loaded with bone graft material in vivo, supported the formation of highly vascularized tissue and endochondral bone without adverse reactions. Demonstrates the use of a novel, complex, and biomimetic meso-scale architecture (Voronoi) in a clinically relevant biodegradable polymer composite for scaffold-guided bone regeneration
[212]	Jonušauskas (2019)	Method: 3D Laser Lithography (3DLL). Material: Organic–inorganic hybrid pre-polymer	A fabrication technique capable of producing meso-scale scaffolds with precisely controlled geometry and sub-micron features	No biological data. The study focused on demonstrating the advanced fabrication capability, showing that meso-scale scaffolds suitable for cell growth can be produced with high precision and throughput. Highlights a high-resolution additive manufacturing technique for creating complex, custom-designed meso-scale scaffolds that could be used in regenerative medicine
[213]	Anandagoda (2012)	Method: Plastic Compression combined with osmotic swelling. Material: Collagen and Hyaluronan (HA)	Osmotic swelling of dry HA within a compressed collagen sheet creates defined meso-scale structures. An HA core forms a central channel (tube), while layered HA creates an open-spiral structure	Morphogenesis via Fluid Dynamics: A novel fabrication method where fluid redistribution driven by HA swelling creates predictable 3D meso-structures. The process was shown to be non-toxic to embedded fibroblasts. This unique method uses the physicochemical properties of natural polymers to self-assemble complex meso-scale architectures without complex machinery
[214]	Ghezzi (2011)	Method: Plastic Compression (PC). Material: Type I Collagen	Creates extracellular matrix (ECM)-like meso-scale characteristics of dense fibrillar collagen (DC) scaffolds (~ 150 µm thick) from highly hydrated gels (~ 3000 µm thick) by expelling > 90% of fluid. This is a meso-scale change in construct thickness and density	Improved Cell Viability and Growth: PC is a cell-safe process. Cells in dense scaffolds showed significantly better survival, metabolic activity, and proliferation compared to cells in thick, hydrated gels, which suffered from cell death in the core. Focused on the immediate cellular response to the PC process, confirming it as a viable method for creating dense, cell-laden constructs without the mass transport limitations of thick hydrogels
[215]	Murphy (2010)	Method: Freeze-drying (Lyophilization) with controlled cooling/annealing. Material: Collagen-Glycosaminoglycan (CG)	A series of scaffolds with controlled mean pore sizes ranging from 85 µm to 325 µm	Larger Pores Optimal for Proliferation: Scaffolds with the largest pores (325 µm) showed the highest cell numbers due to superior cell migration and reduced cell aggregation at the scaffold periphery. Directly investigated the effect of meso-scale pore size, concluding that pores > 300 µm are optimal for cell infiltration and proliferation in these specific CG scaffolds
[216]	Kamranpour (2016)	Method: Gel Aspiration-Ejection (GAE). Material: Type I Collagen	Aspiration of a hydrated gel into a needle compacts it and induces meso-scale fibrillar alignment throughout the scaffold. Creates injectable dense collagen with controllable fibrillar densities (5–32 wt%)	Anisotropy Accelerates Differentiation: The aligned fibrillar structure guided seeded Mesenchymal Stem Cells (MSCs) to align and accelerated their osteoblastic differentiation compared to randomly organized dense collagen. A novel system to produce injectable dense collagen scaffolds with controlled anisotropy, providing both structural and biological cues to direct cell fate

## Titanium and alloys

Commercially pure titanium (cpTi) and its alloys remain the most commonly used materials for load-bearing implants. Their machinability and surface reactivity have allowed for a wide variety of surface modifications at the micro-level. Several techniques have emerged to create meso-scale structures on titanium, each with distinct outcomes in topographical features, geometry, and roughness parameters.

### High-temperature acid etching

Recent advancements have enabled the creation of titanium surfaces with three hierarchical structures spanning the meso-, micro-, and nano-scale via a single-step high-temperature acid etching technique [167]. Commercially pure titanium was etched using 67% sulfuric acid at temperatures ranging from 120 °C to 150 °C for 75 s. This method produced distinct topographies depending on the etching temperature:

- At 120–130 °C: Only traditional micro-scale pits or compartmental structures.
- At 140–150 °C: Meso-scale spikes (10–80 µm diameter) in addition to micro- and nano-scale roughness.

### Morphological characteristics and surface metrics

**Fig. 9:**
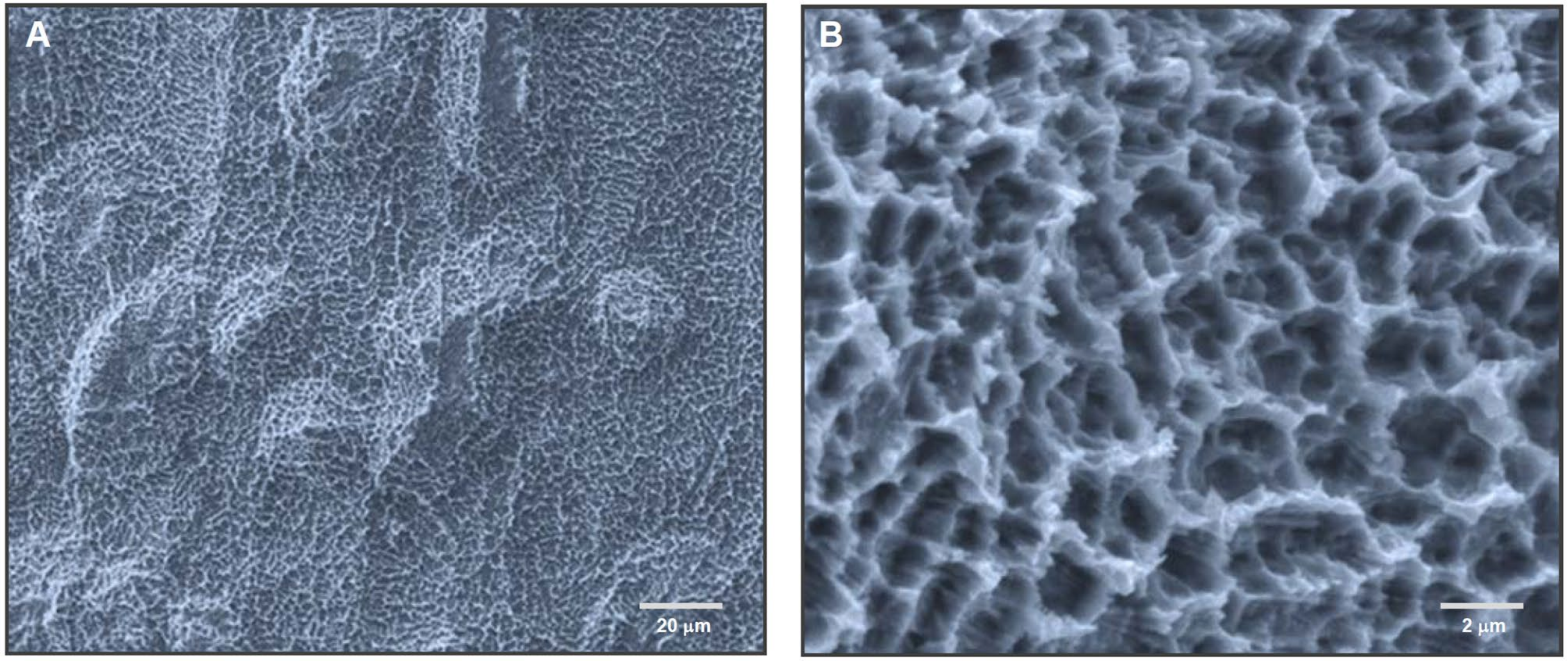
Unprecedented formation of meso-scale topography on titanium by high-temperature acid etching: Toward a three-scale hierarchical titanium surface. SEM images of titanium treated with sulfuric acid at 140 °C, illustrating the successful creation of a hierarchical rough surface incorporating meso-, micro-, and nano-scale features. The low-magnification image (**A**) shows the formation of well-defined meso-scale spike-like structures ranging from 20–70 µm—an unprecedented outcome for acid etching techniques, which traditionally yield only micro-scale pits. The high-magnification image (**B**) reveals conventional acid-etched micropits (~ 1–5 µm), further decorated with polymorphic nano-scale features within the pits and along the pit edges. This three-tiered topography marks a significant advancement in titanium surface engineering, with the meso-scale addition representing the most impactful and transformative feature

Qualitative characterization and quantitative analysis showed (SEM images shown in Fig. 9):

- Average roughness (Ra**)**: Increased sixfold at 140 °C and 12-fold at 150 °C compared to 120 °C-etched surfaces (regular acid-etch-created microrough surfaces).
- Peak-to-valley roughness (Rz)**:** Increased proportionally with temperature.
- Meso-scale spike density: Increased with temperature, plateauing at 140 °C.
- Meso-scale spike size**:** Continued to increase up to 150 °C, with width ranging from 10–80 µm (20–50 µm average) and heights from 10–20 µm.
- Micro-roughness: typical micropits ranging from 0.5–5 µm all over the meso-spikes.
- Nano-textures: defined, polymorphic features, such as nodular, ridge, and compartments features ≤ 400 nm overlaying larger structures.

This acid etching method yields a reproducible, chemically defined, and highly integrated meso-micro-nano topography in a single treatment—an important step toward overcoming the biological limitations of conventional microrough surfaces.

### Biological response and osseointegration

Temperature-controlled surface roughening at the meso-scale enabled a systematic evaluation of osteoblast behavior and osseointegration across a range of surface conditions [167]. As the etching temperature increased, the formation of meso-scale spike structures became more robust and densely distributed. Titanium acid-etched at 120 °C produced only micro-roughness and served as a conventional control, while the 140 °C condition produced the most defined and effective meso-scale topography.

Initial osteoblast attachment was comparable across all temperature conditions, indicating that the introduction of meso-scale structures did not hinder early cell attachment, despite the considerable increase of surface roughness [167]. However, cell morphology and cytoskeletal organization were significantly affected. High-resolution confocal imaging revealed that osteoblasts cultured on high-temperature surfaces exhibited markedly larger spreading areas, elongated perimeters, and extended lamellipodia and filopodia—indicative of more dynamic and robust surface engagement. This finding stands in contrast to conventional microrough surfaces, where osteoblast spreading is typically restricted. Furthermore, the expression of cytoskeletal actin and the focal adhesion protein vinculin increased with temperature, suggesting the formation of stronger and more mature focal adhesions on multiscale roughened surfaces.

Osteogenic differentiation was also markedly enhanced on the high-temperature etched surfaces. Both alkaline phosphatase (ALP) activity and calcium deposition—hallmarks of osteoblast maturation—exhibited a progressive increase with rising etching temperature from 120 to 150 °C [167]. Gene expression analyses confirmed this trend: osteopontin and osteocalcin were significantly upregulated at both early and later culture stages on higher-temperature surfaces. These results demonstrate not only accelerated differentiation but also a higher degree of osteoblastic lineage commitment. Importantly, these effects occurred without impairing early cell attachment or spreading, indicating that the three-scale roughness—especially the addition of meso-scale spikes—overcomes the classic biological trade-off of microrough surfaces, which typically promote differentiation at the expense of proliferation and spreading.

In vivo, the mechanical integration of the implant with bone improved significantly with the incorporation of meso-scale features [167]. Push-in tests at two weeks showed that the 140 °C acid-etched surface exhibited a 2.3-fold increase in mechanical retention compared to the 120 °C group. While both surface roughness and spike density continued to increase from 140 to 150 °C, the peak in osseointegration strength was observed at 140 °C, suggesting an optimal threshold for topographical complexity that balances bone ingrowth with structural interlock.

Regression analyses further supported these findings, demonstrating that key biological outcomes—including osteoconductivity, gene expression of differentiation markers, and biomechanical strength—were strongly correlated with the size and density of meso-scale spikes [167]. These features likely provided expanded surface area and architectural space for cell recruitment and proliferation, compensating for the limitations of microrough surfaces. Ultimately, this high-temperature acid-etching strategy not only introduced effective meso-scale architecture but also resolved the previously intractable biological trade-offs associated with traditional microrough surfaces—representing a significant advancement in implant surface engineering.

### Laser etching

In the past three decades, osseointegration gains have plateaued in recent decades [16, 17, 168–173]. In response, researchers have explored adding larger-scale (10–500 µm) structures to better mimic the size of cells and mineralized tissue subunits, hypothesizing that such meso-scale features can enhance mechanical interlocking with bone and provide cell-guiding cues [174]. Laser etching offers a precise, contamination-free way to create these structures. Unlike grit-blasting (which can leave embedded particles or sharp micro-irregularities), laser ablation modifies titanium without introducing foreign debris. Indeed, laser processing can even thicken the native titanium oxide layer. Key advantages of laser surface modification include the ability to pattern the surface at will (producing organized channels, grooves, grids, or pits) and to adjust feature dimensions reproducibly in the cell-sized range. Recent reviews note that while laser texturing protocols vary, overall outcomes have been promising: most laser-modified surfaces show equal or improved biocompatibility compared to conventional surfaces, and importantly, features on the scale of tens of microns (comparable to cell size) tend to positively influence osteoblast attachment and vitality [174].

### Common laser etching methods for titanium and alloys

A variety of laser technologies have been employed to create meso-scale features on titanium and Ti-alloy surfaces. Pulsed solid-state lasers (especially Nd:YAG or Nd:YVO4 systems) operating in the nanosecond regime are widely used to ablate controlled patterns. For example, Q-switched Nd:YVO4 (355 nm wavelength) pulses of ~ 25 ns can be rastered to carve microchannels without significant heat damage to surrounding material [175]. Using such a setup, parallel grooves ~ 21 µm in width and 18 µm deep were achieved on grade 4 titanium, with minimal melting or residue due to the rapid, high-peak-power pulses. The absence of chemical or grit media means laser-treated surfaces avoid the contamination and embedded particles associated with traditional blasting [175]. On the other end of the spectrum, ultrashort (femtosecond) lasers have been employed to create even finer surface details. Femtosecond infrared lasers (e.g. Yb-based fiber lasers around 1030 nm) can ablate titanium with virtually no thermal diffusion, producing crisp micro-features often decorated with nanoscale ripples or textures from laser-induced periodic surface structures (LIPSS) [174, 176, 177]. Studies show that by adjusting beam parameters and scan strategy, femtosecond lasers can generate complex hierarchical patterns – for instance, a femtosecond laser with 500 fs pulses yielded a surface of micro-scale fissures covered in nano-spikes, with an average roughness *Ra* ~ 0.3 µm [174].

Lasers can be delivered in various modes: direct writing with a focused beam to create linear or circular features, or interference/mask techniques to pattern periodic micro-arrays [174]. The choice of laser and parameters allows tailoring of feature size within the 10–500 µm range, while maintaining control over surface chemistry (oxide formation) and topography at smaller scales.

### Meso-scale morphological features

A common motif is the laser-engraved microgroove or channel. For instance, precise parallel grooves around 20 µm wide and deep have been achieved on Ti surfaces by controlled laser ablation [175]. The commercial *Laser-Lok®* surface is a notable example: it consists of concentric microchannels of approximately 8 µm and 12 µm in width, laser-machined onto the implant collar [178]. These microgrooves are spaced uniformly to create a series of cell-sized troughs encircling the implant neck. Pitted or dimpled surfaces can also be produced by laser pulsing in an array pattern. Researchers created a field of laser-drilled micro-indentations ~ 5 µm in diameter with 15–30 µm center-to-center spacing on titanium, intending to promote soft tissue attachment on abutments [179]. Such laser pits fall within the lower meso-scale and can be arranged regularly, in contrast to random acid-etch pits. On the larger end, meso-scale grooves and grids up to several hundred microns have been demonstrated. Nd:YAG laser was used to cut a crosshatched grid on Ti–6Al–4 V discs, yielding intersecting fissures on the order of 40–140 µm thick [180]. This produced a coarse mesh-like topography intended to improve initial mechanical interlocking and friction upon implant insertion.

Notably, laser machining often inherently yields multi-scale topography: the primary meso-structure (grooves, pits, etc.) may carry superimposed micro/nano textures due to the nature of material ablation and resolidification. For example, laser-formed microchannels (~ 21 µm wide) exhibited nanoscale roughness along their edges, providing additional “sub-micron” topographic cues [175]. Similarly, the Laser-Lok grooves, while orderly, have been shown at high magnification to possess fine-scale uniform ridges within the channels[178]. Depending on laser parameters, one can thus obtain either mono-scale patterns (e.g. purely meso-scale grooves on an otherwise smooth surface) or hierarchical textures that potentially combine meso-, micro-, and nano-scale elements [175].

### Biological response and osseointegration

Laser-etched meso-scale topographies have shown favorable outcomes for osteoblast and osteoprogenitor cell behavior in numerous studies. In general, such surfaces do not inhibit initial cell attachment – a concern for very rough surfaces – but rather often promote cell adhesion and proliferation relative to smooth or conventionally roughened controls [181]. For example, a recent study comparing ground, SLA-etched, and laser-grooved titanium found that human pre-osteoblasts proliferated more on the laser-produced microchannel surface than on the SLA surface over 3–18 days of culture [175]. Similarly, a systematic review of 28 studies concluded that most laser-modified titanium surfaces either improved or maintained osteoblast adhesion and growth compared to standard surfaces [181]. Notably, the presence of meso-scale features commensurate with cell size appears beneficial: surfaces with grooves or pits on the order of 10–50 µm (comparable to cell dimensions) tended to support higher cell viability, spreading, organization, and proliferation.

This may be due to “contact guidance” phenomena, where osteoblasts and stem cells orient along the laser-etched topography. Indeed, in one femtosecond-laser study, osteoblastic cells aligned differently on linear groove patterns versus more isotropic radial patterns, and the isotropic (radial) nano-grooved surface induced greater fibronectin production and osteogenic gene expression [176]. Also, another plausible explanation is that the increased surface area by meso-topography and the relatively rounded or soft-edged micro- and nano-topography commonly seen on the laser-etched titanium do not hinder osteoblastic proliferation as significant as that on microrough titanium surfaces commonly with sharp-edge peaks as shown in Fig. 1.

Studies have focused on optimizing meso-scale pore dimensions to enhance the biological response to laser-textured titanium. In one study, defined pore diameters of 100, 200, and 300 µm were laser-created and combined with grit blasting to generate rough inter-pore surfaces [182]. All pore sizes supported bone remodeling in vivo, but the 100 µm pores exhibited delayed response, suggesting that the 200 µm dimension offered the most favorable balance between cell infiltration and remodeling kinetics. In a separate study, porous titanium structures with controlled porosity (17–58%) and pore sizes up to 800 µm were fabricated, exhibiting naturally rough surfaces formed by partially melted spherical particles [183]. These porous architectures promoted greater osteoblast proliferation compared to smooth titanium. Importantly, cell ingrowth into pores required a minimum pore size of > 200 µm; smaller pores (< 150 µm) were commonly bridged over by cells without infiltration. Together, these findings suggest that laser-engineered pores in the meso-scale range—particularly those ≥ 200 µm—are critical for promoting deep tissue integration and guiding multicellular organization within the implant–bone interface.

Beyond cell adhesion and number, laser-etched surfaces significantly influence osteogenic differentiation of osteoblasts. Pre-osteoblasts on 21 µm laser microchannels exhibited higher mRNA and protein expression of osteogenic markers early as 3 days, compared to cells on SLA or polished surfaces [175]. Likewise, ultrafast laser–induced nanostructures on titanium have been shown to elevate osteogenic genes and lead to more mineralized nodule formation, compared to untreated titanium [176].

Although long-term clinical data remain limited, numerous animal studies and short-term clinical evaluations report improved osseointegration for laser-modified implants. Laser-created roughness often yields higher bone–implant contact (BIC) and stronger early fixation compared to machined or even SLA surfaces [184]. A review on animal studies indicated that laser-modified implants showed superior removal torque than SLA implants [184].

### The laser-Lok™ system: defined meso-scale topography

**Fig. 10:**
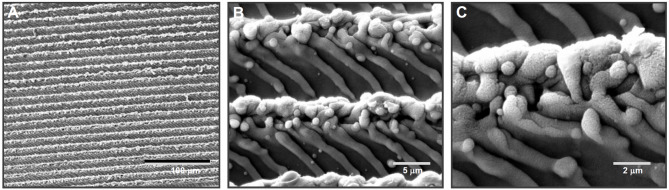
Commercially implemented meso-scale topography: Laser-etched titanium surface with 15 µm channels and hierarchical microstructures. SEM images of a representative laser-textured titanium component (Laser-Lok™, BioHorizons), featuring defined meso-scale surface architecture. **A** Low-magnification image shows monodirectional meso-scale channels with a consistent 15 µm pitch, precision-etched by laser processing. These channels represent one of the few clinically implemented examples of engineered meso-topography in dental implants and abutments. **B** Medium-magnification image reveals the inside of the meso-channels, where superimposed woven oblique micro-ridges and nodular features are observed—likely formed as secondary roughness due to laser-induced resolidification. **C** High-magnification image highlights irregular micro-nodules populating the channel crests, adding further microscale complexity to the meso-groove foundation. This example illustrates how meso-scale structures (in the 10–50 µm range) can be systematically introduced to implant surfaces using laser technology, offering contact guidance for cells, guided tissue response, and an expanded interfacial area

One of the most clinically studied implementations of meso-scale topography is the **Laser-Lok™** system from BioHorizons—an example of precision laser-etched implant collars and abutments. The Laser-Lok concept was designed to achieve dual-affinity integration: osseointegration in combination with a soft tissue connective-tissue. These feature regular grooves approximately 8–12 μm in width—designed to guide cell orientation, promote connective tissue attachment, and reduce epithelial downgrowth. While primarily aimed at soft-tissue stabilization, histologic and clinical studies show superior peri-implant collagen organization and reduced crestal bone loss (~ 0.44 mm over two years) compared to machined collars [185]. A particular study using Laser Lok™ abutment described as a distinct morphology consisting of monodirectional mesoscale channels (15-μm pitch) and woven oblique micro-ridges formed within the channels [178] (Fig. 10**).** The micro-ridges formation is supposed to be a secondary roughness made by the consequence of resolidation as discussed earlier. Quantitative surface roughness was not presented in this study.

Evaluations of Laser-Lok implants have consistently reported favorable outcomes: greater bone-to-implant contact, less marginal bone loss, and healthy peri-implant soft tissues, relative to traditional surfaces [186, 187]. However, a majority of the reports are from clinical setting and the systematic in vitro experiments using osteoblast or detail analysis of bone histology and biomechanical testing remain to be conducted.

### Summary

Laser etching presents a promising avenue for introducing meso-scale topography on titanium surfaces in a clean, precise, and reproducible manner. Unlike conventional microroughening, laser texturing allows customization of feature geometry to match cell-scale dimensions, potentially overcoming the biological trade-off between osteoblast proliferation and differentiation seen with micro-scale roughness. It enables integration of hierarchical architecture—meso-, micro-, and nano-scale features. Clinical systems like Laser-Lok™ provide early evidence of its translational potential, though further systematic in vitro and biomechanical studies are warranted. Overall, laser-based meso-texturing is a valuable, underutilized tool for engineering next-generation implant surfaces with optimized biological and mechanical performance.

### 3D printing

#### Overview: additive manufacturing

Additive manufacturing (AM) of titanium (particularly Ti–6Al–4 V) for implants is most commonly done via powder bed fusion techniques, notably selective laser melting (SLM, also known as laser powder bed fusion) and electron beam melting (EBM). These processes build implants layer-by-layer from metal powder, inherently yielding rough surfaces due to partially melted or sintered particles adhering to the surface [188, 189]. SLM uses a focused laser and offers finer resolution, whereas EBM uses an electron beam in vacuum and tends to produce coarser surfaces and larger melt pools. As a result, EBM parts generally have a rougher “as-built” surface finish than SLM parts, often requiring aggressive post-processing (machining or polishing) to achieve smooth surfaces. For example, EBM-fabricated Ti–6Al–4 V components commonly show *Ra* roughness on the order of tens of microns (20–50 µm under typical settings) without post-treatment. SLM surfaces are somewhat smoother but still significantly rough: the surface arithmetic average height (Sa) of as-printed SLM Ti implants is typically around 9–20 µm [188], meso-scale, i.e., an order of magnitude higher than conventionally grit-blasted and acid-etched (SLA) implants which have Sa ≈1–2 µm [145].

#### Method of creating meso-scale roughness: during printing or through post-processing

Surface topography can be introduced either during the printing process or through post-processing. During printing, designers can incorporate porous lattices or raised features on substrate surfaces. For instance, one study 3D-printed mesoscale “spikes” (sprouts) about 500 µm tall on a titanium surface [190]. These CAD-designed protrusions aimed to increase roughness and surface area without additional post-processing. Likewise, SLM and direct metal laser sintering (DMLS) can fabricate interconnected porous surface layers by partially melting powder to achieve a designed pore network [78, 191, 192].

Post-processing techniques are also widely used to modify surface topography after printing. Common methods include abrasive blasting or shot peening (to impart a uniform micro-rough texture), acid etching (to dissolve surface layers and create micropits/nanostructures), and laser ablation (to carve grooves or patterned features). For example, acid etching of SLM or EBM surfaces can remove loosely attached small powder particles while leaving behind the larger scale roughness features [193]. Industry approaches after additive manufacturing often involves removing the native roughness and re-engineering a new roughness that mimics traditional implant surfaces [189].

#### Meso-scale surface feature characteristics

Additively manufactured titanium often exhibits multi-scale roughness comprising: (a) microroughness from melt pool solidification and fine powder (~ 1–20 µm features), and (b) meso-scale asperities from partially fused or sintered particles (~ 10–100 µm particles) or intentionally designed pores and protrusions[188, 189]. In SLM/EBM parts, it is typical to see spherical or irregular Ti particles tens of microns in diameter attached to the surface, creating a high surface roughness (Sa ≈ 9–20 µm) as noted above. The peak-to-valley height (Ra) on as-built surfaces can be even larger; one experiment reported Ra on the order of 40–65 µm for printed rough surfaces, versus < 10 µm for polished ones [190].

Beyond these stochastic features, controlled porous architectures fall in the meso-scale range and are a key advantage of 3D printing. Porous lattice or foam-like surfaces with pore sizes of 100–500 µm can be built into dental or orthopedic implants to promote bone ingrowth. Another study printed a trabecular-like Ti lattice for a femoral implant (pore ~ 500 µm); the porous surface roughness (Sa) exceeded 15 µm, significantly higher than a solid acid-etched surface (~ 2 µm), and facilitated bone ingrowth into the pores [188]. In another experiment, SLM was used to print ~ 500 µm tall tetrahedral spikes on a Ti sample to simulate a very rough surface [190]. These printed spikes increased the Ra to ~ 6–10 µm (versus ~ 1 µm polished) and Rz up to ~ 65 µm.

**Fig. 11:**
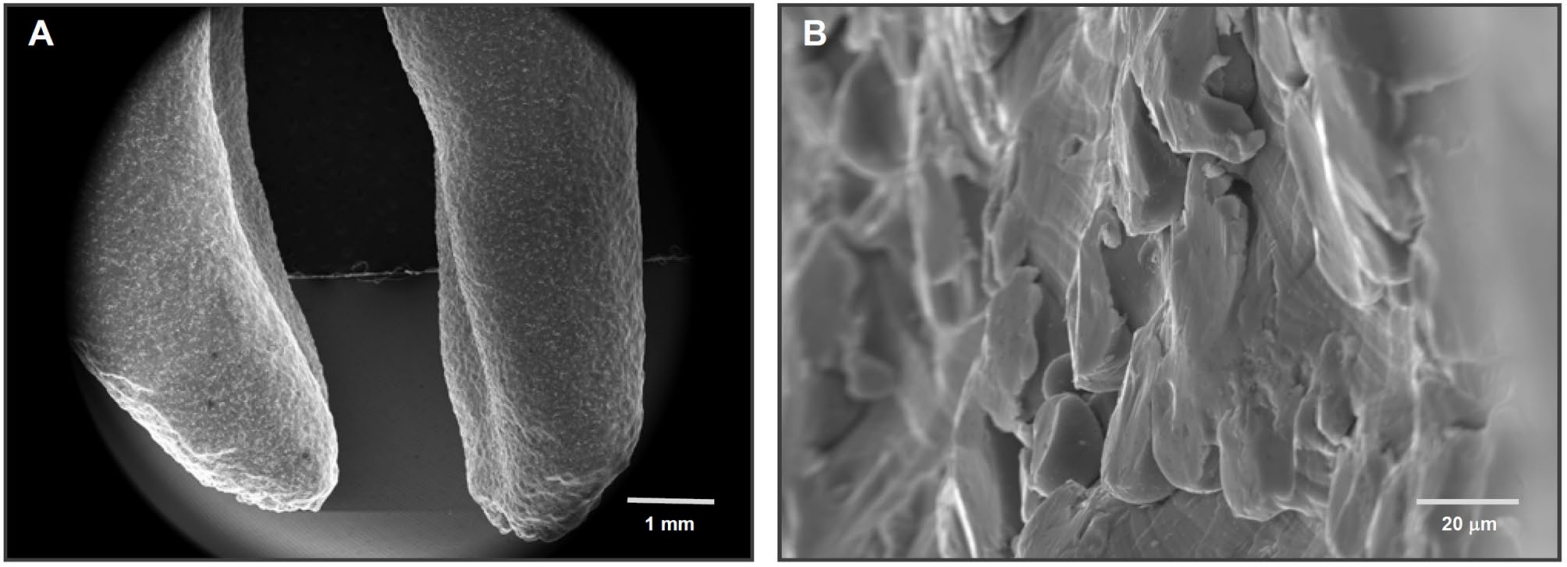
Stochastic meso-scale surface topography on additively manufactured titanium. SEM images of a Ti6Al4V specimen fabricated via selective laser melting (SLM) based on a digital scan of an extracted human molar. The as-printed surface demonstrates irregular, nonuniform meso-scale topography with randomly fused and partially melted titanium particles ranging from 10–100 µm in diameter. These structures reflect the intrinsic nature of AM processes and represent a distinct departure from conventionally machined or etched surfaces. Unlike deterministic micro-roughening, this meso-scale architecture offers a complex spatial landscape that may enhance mechanical interlocking and cellular accommodation

In terms of roughness parameters, conventional blasted/etched implant surfaces are “moderately rough” (Sa ~ 1–2 µm) [19, 144]. By contrast, as-printed additively manufactured surfaces often lie outside this range – they are much rougher (Sa in the high single or double digits). Post-processing can modify these values: Usually it tempers the surface roughness depending on the condition. Grit blasting with coarse media (250–500 µm alumina, for instance) yields roughness on the order of ~ 5–10 µm Ra, while acid etching superimposes sub-micron pits on top of whatever micro/meso texture is present [189]. It’s noteworthy that acid etching tends to remove small loosely-bound particles from AM surfaces but not the larger anchor-like particles. Thus, an acid-etched SLM surface may still exhibit meso-scale “bumps” (surviving large particles) protruding from a newly generated micro/nano-textured base. This highlights that AM can produce hierarchical topographies depending on the method, condition, post-processing, and a combination of these. Figure 11 exemplifies this phenomenon: an SLM-fabricated Ti6Al4V surface produced from a digital scan of an extracted molar. The resulting surface exhibits irregular, nonuniform meso-scale topography characteristic of AM—providing a visual demonstration of the capacity of this technology to generate stochastic, yet functionally relevant roughness.

#### Advantages and challenges

Porous and complex surfaces enabled by 3D printing offer clear advantages: the ability to customize porosity and roughness to foster bone integration and to match patient anatomy. The design freedom means that load-bearing implants can have solid cores for strength and porous, bone-mimicking surfaces for osseointegration – geometries impossible to create with traditional machining. Studies have shown that porous additively manufactured implants can be fabricated from titanium. For example, a multi-rooted dental implant with a 3D-printed porous surface was successfully fabricated [191].

However, there are several challenges and trade-offs in fabricating these surface features:

- *Reproducibility and Precision:* Achieving consistent meso-scale topography on every implant can be difficult. The additive manufacturing (AM) process has inherent variability – factors like powder size distribution, laser power, scan strategy, and part orientation can all affect surface roughness. Research shows that even with identical machine settings, surface roughness of SLM Ti parts can vary, and different faces of the same part (vertical vs. angled surfaces) yield different Ra values [194]. In AM, surface roughness arises from two main sources: "primary roughness" from melt pool solidification and "secondary roughness" from partially fused powder particles. Electron Beam Melting (EBM), which typically uses larger powder particles (50–150 µm) than Selective Laser Melting (SLM; 10–60 µm), tends to produce rougher surfaces. This is due in part to EBM’s broader melt pool and high scanning speeds (> 1000 m/s), which increase the likelihood of large particle adherence and uneven surface morphology.
  While CAD-designed features (e.g. a lattice unit cell) are repeatable by design, the *as-built* reality may differ due to partially melted particles and local thermal effects. Ensuring each porous structure is fully open and within the target 100–500 µm size range requires tight process control. Porosity control in AM is non-trivial – for instance, increasing laser energy can eliminate unintended pores and smooth the surface but might overshoot the porosity target. Conversely, reducing energy or scan speed increases as-built roughness and intentional pore size but risks lack-of-fusion defects. Optimizing these parameters is a complex, interdependent problem. Post-fabrication inspection (e.g. micro-CT to confirm pore sizes, surface profilometry for Ra) is essential to verify consistency.
- *Mechanical Strength and Fatigue:* Introducing surface porosity or large roughness features inevitably impacts the mechanical properties of the implant. Meso-scale surface features act as stress concentrators – they can reduce the effective load-bearing cross-section and serve as initiation sites for cracks [190]. In a controlled study, Ti–6Al–4 V tensile bars with 3D-printed 500 µm “sprout” features on their surface showed ~ 2% lower ultimate strength and reduced ductility compared to smooth bars. The roughest samples (with dense 500 µm protrusions) had about a 2–5% drop in tensile strength and slightly lower elongation. Fatigue performance is even more sensitive: high-cycle fatigue life can be drastically shortened by as-built rough surfaces, which is why critical load-bearing implants often undergo surface polishing in fatigue-sensitive areas. There is a balance to strike – for example, retaining a porous structure for osseointegration but applying hot isostatic pressing (HIP) and machining to smooth internal defects can restore fatigue strength. In sum, while porous or rough surfaces improve biological fixation, they must be engineered not to unduly compromise mechanical reliability.
- *Post-Processing Limitations:* Achieving uniform surface treatment on complex 3D-printed implants presents significant challenges. Intricate internal architectures—such as lattice pores or channels found in porous spinal cages or acetabular cups—are often inaccessible to conventional mechanical methods like polishing or grit blasting [189]. While chemical etching can reach into complex geometries, it may produce uneven effects: smaller features risk being completely dissolved, whereas larger structures may remain largely unaffected. Laser ablation offers high precision and can introduce tailored patterns (e.g., 200 µm grooves for guided cell migration), but it is limited by its line-of-sight nature and cannot treat undercut or recessed areas. Moreover, laser processing is time-intensive, further constraining its practicality for large or complex surfaces.

#### Biological response and osseointegration

Unlike traditional subtractive or coating-based techniques, 3D printing allows precise spatial design and reproducibility, making it not just a means of producing better implants, but also a powerful experimental model to test and refine the ideal morphological cues that guide osseointegration. As such, 3D-printed titanium surfaces provide a valuable interface between material science and bone biology—allowing researchers to systematically evaluate how topography influences cellular and tissue-level responses. The growing body of biological literature emerging from these studies offers insights, implications, and even design rules that can inform future implant innovations beyond 3D printing itself.

Early experiments on 3D-printed Ti implants found that as-built SLM surfaces (with ~ 10 µm Sa) can enhance osteoblast activity relative to smooth polished Ti. For example, significantly higher osteoblast proliferation and alkaline phosphatase (ALP) activity were found on an SLM rough surface compared to a machined surface in vitro [195]. On the other hand, in vivo studies show that coarse AM surfaces can confer significant benefits in early osseointegration and mechanical fixation. A rabbit study compared SLM-fabricated dental implants (as-printed rough surface) to conventional sandblasted/acid-etched implants. Histologically, both surfaces achieved equivalent bone-implant contact, but the SLM implants exhibited a remarkable early advantage in fixation strength [188]. Finite element analysis in that study further suggested that the irregular topology of the SLM surface helped distribute interfacial stress, reducing strain concentrations in the adjacent bone under load. These findings illustrate a key point: while meso-scale roughness per se didn’t accelerate cell proliferation at the cellular level, it provided a mechanical scaffold for rapid load-bearing integration in vivo. The early stability granted by such interlocking could be clinically important for immediate or early loading of implants. Porous meso-structures have shown even more dramatic biological effects. The multi-rooted SLM implant with a porous surface mentioned earlier not only had greater bone ingrowth, but bone had infiltrated deep into the pore structure and even in between the “roots” of the implant [191]. This ingrowth translated to much higher push-out force and torque resistance, indicating true osseointegration via bone ingrowth as opposed to just surface apposition.

## Summary

3D printing has opened new avenues to engineer implant surface topography across length scales. In particular, meso-scale structuring (10–500 µm), such as porous networks or large asperities, are benefited most and readily produced by SLM/EBM and can effectively increase implant surface area and enable bone ingrowth. The morphological outcomes include roughness values (Ra) an order of magnitude above traditional implants and pore sizes tailored to bone biology. Key advantages are improved initial mechanical fixation and osteoconductivity, as demonstrated by higher removal torques and bone ingrowth in vivo. The major challenges lie in controlling these features without compromising mechanical integrity and consistency – excessive roughness can reduce fatigue life, and process variations can affect reproducibility. Ongoing research is focusing on achieving an optimal multiscale surface (meso/micro/nano) that leverages 3D printing’s design freedom while satisfying mechanical and clinical requirements. Early experimental and preclinical evidence suggests that when properly optimized, additively manufactured meso-scale topographies can significantly improve the integration of titanium implants with bone, accelerating the healing and success of dental and orthopedic implants.

## Zirconia and ceramic materials

Ceramic implants, particularly zirconia (ZrO₂), are gaining popularity due to their esthetics, chemical stability, and biocompatibility. However, their inert and brittle nature makes conventional etching and blasting methods less effective. As a result, alternative strategies are employed to achieve surface roughness or texture, distinct from titanium. Most studies reviewed in this study focused on yttria-stabilized tetragonal zirconia polycrystal (3Y-TZP) used for dental implants [196–198].

Laser etching, extensively used for titanium to form meso-scale features, is increasingly applied to zirconia with distinct advantages and challenges. While both materials can be laser-structured into multi-scale topographies, zirconia requires higher precision to avoid microcracking and phase transformation due to its ceramic nature. In contrast to titanium, zirconia’s inert chemistry means laser ablation must rely more on physical structuring than on inducing surface reactivity. Nevertheless, both materials benefit from laser-controlled creation of meso/micro/nano hierarchical surfaces to enhance biological performance.

### Laser etching

Nanosecond pulsed solid-state lasers and ultrashort pulsed lasers have been used to create meso-structures, periodic grooves or pits in the tens-of-microns range. For example, laser-etched grooves ~ 45–125 µm wide and 23–50 µm deep were achieved on 3Y-TZP disks using an Nd:YAG system [196]. In particular, ultrashort pulsed lasers: Femtosecond lasers have also been applied to zirconia. These can create finer micro/nano-scale textures superimposed on meso-features. An integrative review reports that femtosecond laser irradiation (e.g. ~ 10 nJ pulses at 80 MHz) produces multi-scale roughness (micro + nano) on zirconia, leading to enhanced cell response [199].

### Morphological features

Laser etching allows controlled “meso-rough” patterns on zirconia in the 10–500 µm scale. Common outcomes are arrays of grooves, ridges, or pit clusters: for instance, parallel grooves ~ 50 µm apart and ~ 6–8 µm deep with smoother curved profiles have been reported [197]. These primary grooves are often accompanied by secondary roughness: micro-scale valleys 1–10 µm wide and < 3 µm deep, and nano-scale nodular textures < 0.5 µm. Such hierarchical surfaces (meso/micro/nano) were demonstrated via solid-state laser “sculpturing” of zirconia [78]. The average roughness (Ra) of laser-roughened zirconia can be on the order of ~ 1–2 µm or higher, often several-fold greater than a machined smooth surface. For example, such meso/micro/nano-textured zirconia had an Ra ~ 5 × higher than smooth zirconia[186]. Importantly, laser processing does not change the underlying zirconia chemistry, avoiding compromises in phase or composition [196].

Controllable texturing includes Nd:YAG laser creating grooves on zirconia with widths ~ 125 µm, ~ 85 µm, and ~ 45 µm (depths ~ 50 µm down to 23 µm) by varying the laser settings [175]. By adjusting pulse energy, scan speed, and number of passes, one can modulate the surface roughness (Ra) of a laser-textured ceramic from ~ 3 µm up to ~ 19 µm [200]. Slower scans or multiple passes result in deeper ablation and thus larger meso-scale features. The laser-formed “valleys” between ridges are on the order of tens of microns.

**Fig. 12:**
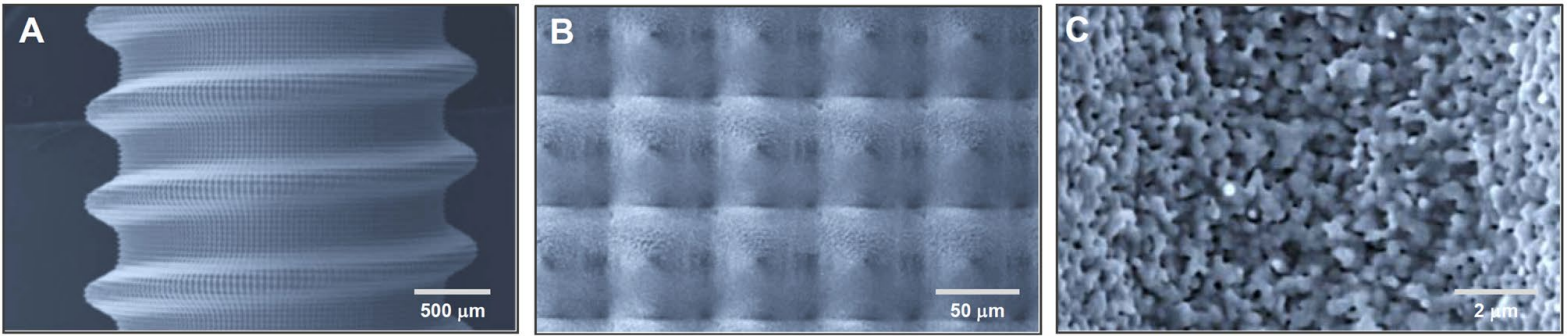
Cactus-inspired meso-structured zirconia surface fabricated by laser etching. SEM images of a prototype Y-TZP dental implant surface show a tri-level hierarchical topography generated via laser ablation. **A** Low-magnification image reveals the macro-scale implant geometry, including threads, decorated with regularly spaced cactus-like meso-scale protrusions (~ 50–80 µm in diameter)., **B** Medium-magnification image provides a top-down view of the meso-scale spikes, **C** High-magnification image highlights the micro- and nano-scale features superimposed on the spike structures, including trabecular bone-inspired nanonodules forming a dense, interwoven texture. This surface architecture mimics natural bone hierarchy and offers enhanced potential for mechanical interlocking, osteoblast guidance, and stable osseointegration

In a detailed optimization study, a biomimetic "cactus-inspired" topography was created on Y-TZP by precisely controlling energy density, pulse duration, and scan interval [201, 202]. The laser-processed zirconia exhibited uniformly spaced meso-scale protrusions (~ 50–80 µm in diameter) surrounded by micro- and trabecular nano-scale textures, forming a tri-level hierarchical surface (Fig. 12). Scanning electron microscopy revealed spike-like elevated structures with radial ridges and fine peripheral undulations. Notably, laser ablation did not cause surface melting or microcracks, preserving structural integrity and avoiding undesirable phase transformation of zirconia. These findings demonstrate that solid-state laser systems can generate reproducible and chemically stable meso-scale textures, further enriched by secondary micro- and nano-scale features.

### Biological response and osseointegration

A valley size around ~ 20 µm is comparable to the diameter of osteoblastic cells (~ 10–30 µm) when they are in their initial spreading stage, meaning cells can physically sit within and bridge these laser-cut grooves [70, 200]. This scale of topography is thought to facilitate mechanical interlocking of new bone and provide spatial cues to cells [101]. As anticipated this way, overall, osteogenic cell response tends to improve on laser-etched zirconia compared to smooth surfaces. Osteoblast-like MC3T3 cells on fiber-laser grooved zirconia showed elongated morphologies aligned with grooves and more filopodial extensions, whereas on smooth zirconia they spread more randomly [203]. Several studies report increased osteoblast proliferation and differentiation markers on laser-textured zirconia [203]. Likewise reported that bone-related genes (Collagen I, Osteopontin, Osteocalcin, BMP-2) were 7–25 × upregulated in rat osteoblasts cultured on hierarchically rough (laser-engraved) zirconia versus smooth controls [197]. More importantly, cell proliferation was not negatively impacted unlike on microrough titanium surfaces. This suggests that laser-created meso/micro/nano features strongly promote the both osteogenic proliferation and differentiation. The overcome osteoblastic dichotomy is probably due to the increased surface area by the meso-texturing that compensated the adverse effect of the increased roughness.

Several experiments in animal models indicate that laser-etched zirconia surfaces achieve improved bone integration (osseointegration) compared to smooth surfaces. Laser-roughened zirconia implants had significantly higher bone–implant contact (BIC) and required higher removal torque to detach, compared to smooth implants [203]. In fact, the removal torque and BIC for laser-textured zirconia approached levels typically seen with roughened titanium implants. Similarly, laser-etched zirconia exhibited the strength of osseointegration *double* that of machined zirconia[197]. Even at these early times, histology/SEM showed mineralized bone tissue growing intimately around the laser-rough zirconia, whereas smooth implants had less bone coverage. Encouragingly, the performance of laser-modified zirconia implants has begun to approach that of conventional Ti implants. In a dog study, zirconia implants treated with a laser attained ~ 48% BIC on average, which was statistically comparable with the ~ 62% BIC for similar titanium implants in the same model [199]. This suggests that with an optimized laser-created topography, zirconia can osseointegrate nearly as well as Ti in vivo.

Utilizing the above-mentioned “cactus-inspired” meso-scale spiked zirconia surface, the researchers optimized the meso-scale topography. They achieved 60 µm diameter spikes (20–80 µm tall) in a lattice across the zirconia, with a superimposed 200–300 nm trabecular texture, to mimic the multi-scale roughness of cactus spikes and bone trabeculae [201]. The 40 µm-high spikes were found optimal, increasing surface area ~ 2.6-fold and roughness Sa to ~ 7–18 µm. In vivo push-in tests showed the 40 µm spiked/nanotextured implants had 8 times greater integration strength than polished zirconia, and even outperformed micro-rough titanium by a wide margin. This study also demonstrated that the cactus surface significantly mitigated or negated the adverse events of biological trade-off by significantly increasing the initial cell attachment and proliferation compared to the microrough titanium surface.

A unique finding has been surfaced about the protein adsorption and initial biological environment associated with meso-scale topography. Immediately upon implantation, the implant surface becomes the site of protein adsorption and blood clot formation—critical events that establish the provisional matrix and influence subsequent cell recruitment. Although the effect of surface topography of implants on these has not yet been widely investigated, recent computational fluid dynamics (CFD) models provide compelling insights [204]. Three surface textures—a smooth amorphous surface, a nano-trabecular design, and a hybrid meso-spike with nano-trabeculae—were compared under simulated blood flow conditions. The hybrid surface reduced blood velocity at the interface by three- to four-folds compared to the nano-only and smooth surfaces, resulting in markedly greater fibrinogen retention at the implant–tissue interface. These data strongly suggest that meso-scale features can physically trap blood and proteins, effectively enhancing clot stabilization and cell recruitment through unique fluid-dynamic mechanisms.

### Summary comparison with Titanium

While laser etching on both zirconia and titanium enables fabrication of meso-scale features, the process must be adapted for material-specific characteristics. Titanium allows for deeper texturing with minimal structural risk, whereas zirconia requires finer tuning to prevent phase changes and surface cracks. Despite these challenges, laser-structured zirconia—with properly optimized meso/micro/nano topography—can now rival or even exceed the osseointegration of microrough titanium, offering a promising platform for esthetic and biofunctional ceramic implants.

### 3D printing

#### Comparison with titanium additive manufacturing

Additive manufacturing (AM) of zirconia shares a common goal with titanium 3D printing: achieving controlled meso-scale topographies and complex geometries tailored for osseointegration. However, the two materials present contrasting fabrication challenges. While titanium can be directly printed using laser-based powder bed fusion methods like selective laser melting (SLM) or electron beam melting (EBM), zirconia must be printed indirectly via slurry- or resin-based methods such as stereolithography (SLA/DLP) or inkjet printing, followed by high-temperature sintering. Titanium offers more flexibility in producing overhanging features and fully dense structures in a single step, whereas zirconia printing is constrained by the risk of shrinkage, cracking, and phase instability during post-processing. Nevertheless, AM offers a unique opportunity to introduce meso-scale roughness or porosity into zirconia, which cannot be achieved via conventional blasting or etching due to its chemical inertness.

### Method and morphology

Various 3D-printing methods have been explored for zirconia. One example is nanoparticle ink-jet 3D printing, used to produce dental implants with an integrated lamellar porous surface layer about 30–40 µm thick [205]. Another is digital light processing (DLP) stereolithography, which builds parts layer-by-layer from a photosensitive zirconia slurry [206]. Printed parts undergo debinding and sintering to achieve full density in the core, while intentionally retaining a controlled porous or rough surface. DLP-printed zirconia discs were tested “as-sintered” (no post-finishing), leveraging inherent layer microtexture. Processing parameters like layer thickness, build orientation, and sintering profile are tuned to yield meso-scale features without compromising structural integrity [205].

### Biological effects

Additively fabricated zirconia surfaces have demonstrated favorable osteogenic responses. The lamellar-porous 3D printed zirconia implants induced elongated osteoblast morphology aligned with the pore direction and supported robust osteoblast proliferation, long-term adhesion, and mineralized matrix deposition in vitro [205]. In fact, the osteoblast response on 3D-printed zirconia (viability, ALP activity, Collagen I expression) was comparable to or better than that on conventional sandblasted/acid-etched titanium in one study [206]. This indicates that even without additional post-sinter treatments, the AM zirconia’s microtexture is bioactive enough to trigger osteogenic cell functions. Gyroid-form zirconia scaffolds (pore ~ 600–800 µm) showed excellent cell infiltration in vitro, and analogous ceramic triply periodic minimal surface (TPMS) scaffolds with ~ 70% porosity significantly enhanced osteogenesis and angiogenesis in a rabbit bone defect model [207]. Together, these findings highlight that 3D printing can create meso-scale textured zirconia implants that maintain high strength while improving bone-cell guidance and osseointegration.

### Summary comparison and perspective

Compared to titanium, the additive manufacturing of zirconia is more dependent on post-print thermal processing and requires finer control of powder formulation, binder removal, and sintering to avoid microcracking and retain mechanical integrity. While titanium AM allows direct formation of dense, mechanically robust structures with roughened surfaces, zirconia AM is best leveraged for designing controlled porosity or meso-scale surface patterns through printing and sintering sequences. Despite these differences, both materials benefit from the freedom of AM to create functionally graded or hierarchical textures that promote osseointegration. Notably, 3D-printed zirconia has begun to demonstrate osteogenic performance that rivals or even exceeds traditional titanium implants, especially when used to produce complex, scaffold-like topographies. This suggests that additive manufacturing may play a critical role in advancing zirconia as a next-generation alternative to metal-based implants.

## Polymeric and natural meso-scale scaffolds: Beyond titanium, toward deeper insight

Although the advances discussed in this section extend beyond titanium or other metallic implant materials, they provide critical insight into future implant design. Like 3D printing, scaffold engineering is grounded in mathematical modeling and structural optimization, offering a powerful platform for studying how surface geometry at the meso-scale influences cellular behavior. These engineered constructs, whether made from natural or synthetic polymers, serve as controlled models to probe and maximize osteoblast recruitment, bone matrix formation, and overall osseointegration. The findings from such scaffold-based systems help define design strategies that may be translated into next-generation surface architectures for titanium implants.

### Rationale for meso-scale scaffold design

Topography in particular pore size and geometry in this range determine cell infiltration, vascularization, and ultimately bone formation. Studies have shown that multi-scale porosity is ideal: smaller pores (~ 50–100 µm) increase surface area for initial osteoblast attachment, while larger pores (~ 200–400 µm) promote deeper nutrient diffusion and blood vessel in-growth [208]. In fact, pores exceeding ~ 300 µm are often necessary to enable robust vascularization and osteogenesis in vivo [209]. Conversely, very small pores without sufficient spacing can create hypoxic microsites that induce an endochondral (cartilage-mediated) route of ossification [78, 208]. Thus, the rationale for meso-scale topographical cues is to strike a balance – providing a scaffold architecture that supports cell seeding and osteogenic differentiation, while also permitting vascularized bone tissue ingrowth. Additionally, geometrical cues at this supra-cellular scale can direct tissue organization. For example, introducing ~ 100–500 µm patterned features in a scaffold can impose contact guidance and confinement on cell clusters, aligning cells and newly deposited matrix along the feature orientation [210]. Meso-scale boundaries (~ 500 µm width) could override micro-scale cues to guide mesenchymal stem cell alignment and collagen fiber [210]. Overall, incorporating meso-scale design elements into biomaterial scaffolds is motivated by the need to recapitulate bone’s native 3D microenvironment – enhancing cell colonization, guiding tissue architecture, and ensuring channels for vascular invasion – all of which are crucial for successful bone regeneration.

### Fabrication techniques and morphologies

A variety of fabrication strategies have been developed to introduce meso-scale topographies (10–500 µm features) in natural and synthetic scaffolds. Common methods include traditional foam templating approaches and advanced additive manufacturing [69]:

- *Porogen leaching and foaming:* Water-soluble salt or sugar particles (100–500 µm) can be mixed into polymers like poly(lactic-co-glycolic acid) (PLGA) or polycaprolactone (PCL), then leached out to yield an open-pore network. This produces random, interconnected pores on the order of the porogen size (typically a few hundred microns) that support cell infiltration and nutrient flow [208]. Gas-foaming and freeze-drying (lyophilization) techniques similarly create sponge-like scaffolds with meso-scale voids; for instance, lyophilized collagen or chitosan gels often exhibit 50–200 µm pores which can be aligned by directional freezing. These straightforward methods yield high-porosity matrices, though pore size and geometry are somewhat stochastic.
- *Additive manufacturing (3D printing):* Emerging 3D printing technologies allow precise control over scaffold architecture at the meso-scale. Melt extrusion printers can lay down filaments in defined patterns, creating regular arrays of pores (typically 200–500 µm) throughout the scaffold. This enables designs such as orthogonal lattices or biomimetic Voronoi structures with porosities > 70%, tailored to match bone’s mechanical and transport requirements [211]. Advanced microscale printing methods push resolution further: for example, femtosecond laser two-photon lithography can fabricate free-form scaffolds with sub-μm precision while building up to millimeter scales [212]. This “mesoscale laser 3D printing” produces scaffolds that integrate nano/micro-scale features (fine fibers, ridges) with overall meso-scale dimensions, potentially allowing cell-instructive surface details within a larger construct.
- *Self-Assembly and Compaction Techniques:* Natural polymer matrices like collagen and hyaluronic acid can be structured at the meso-scale through physical processing. Plastic compression (PC) of collagen hydrogels is one example: applying a uniaxial load expels fluid from a cell-seeded collagen gel, rapidly compacting it into a dense collagen sheet (~ ∼5–20% of original thickness) [213]. The resulting scaffold has an interconnected fibrillar network at high collagen density with ECM-like meso-scale characteristics, improved stiffness, and minimal long-term deformation. Layering such collagen sheets can form 3D constructs with controlled ~ 100 µm thickness per layer. An elegant twist on this approach uses osmotic gradients to create channels: inserting a strip of dry hyaluronic acid into a compressed collagen sheet causes local swelling and “dehydration” of the surrounding collagen, forming open tubular microchannels and new interfaces within the scaffold [213]. These emergent hollow structures in meso-scale (tens to hundreds of microns in diameter) resemble vascular or neural conduits, and can be produced in predictable patterns (e.g. spirals, concentric tubes) without harsh processing [214]. Finally, micropatterning and lithography have been used to fabricate grooved or pillar-like topographies in hydrogels (e.g. photopatterned protein lines in a otherwise non-adhesive hydrogel) [210]. While often at the smaller end of the meso range (~ 10–50 µm feature width), such patterns can guide cell placement and multicellular assembly, essentially “pre-organizing” tissue structure in the scaffold.

Each of these fabrication approaches produces distinctive morphologies at the 10–500 µm scale. Collectively, they provide a toolbox for engineering scaffold architecture to investigate and optimize bone-regenerative cues.

### Osteogenic cell responses and bone regeneration outcomes

Scaffolds with appropriately sized pores enhance initial cell seeding by offering more surface area and trapping cells within the matrix. For example, collagen–glycosaminoglycan scaffolds with ~ 95% porosity and mean pore ~ 100 µm achieved higher seeding efficiency and osteoblast attachment, especially when compared to denser, smaller-pore matrices [215]. However, long-term cell infiltration and proliferation require larger void spaces. Uniformly large pores (> 300 µm) have been shown to permit cells (and progenitors) to migrate deeper and populate the scaffold core, whereas scaffolds with predominantly sub-100 µm pores often see cell accumulation only near the surface [209].

Perhaps the most notable findings are that meso-structured scaffolds can accelerate or enhance the differentiation of stem cells into bone-forming cells. Dense fibrillar collagen scaffolds produced by plastic compression are a representative case – these matrices, by virtue of their ECM-mimetic architecture and stiffness, have been found to stimulate osteoblastic differentiation of pre-osteoblasts and mesenchymal stem cells in vitro [214]. Plastically compressed collagen gels (with meso-scale fiber density and pores) supported higher cell metabolic activity and upregulated osteogenic markers compared to traditional dilute collagen gels. In the injectable aligned collagen gels, human MSCs showed more rapid alkaline phosphatase expression and mineralization when the scaffold combined high collagen density with aligned fibrils, as opposed to random-fiber controls [216]. The induced anisotropy at ~ 100–200 µm scale in these gels effectively provided contact guidance to the cells, promoting a differentiated, osteoblast-like phenotype.

Meso-scale topography can also modulate the quality and orientation of extracellular matrix produced by osteogenic cells. Imposing geometric constraints like ~ 500 µm channels or strips can align the secreted matrix; although osteoblasts do not require aligned fibers as tendon cells do, a degree of spatial organization (e.g. oriented collagen) might favor the formation of lamellar bone structure [210]. When meso-scale boundary conditions were introduced, the collagen I matrix produced by MSCs was more aligned, even though key osteogenic gene expression was not significantly changed [210]. This suggests that meso-features can guide tissue architecture independently of biochemical differentiation cues, which may enhance the mechanical functionality of regenerated bone.

Meso-porous scaffolds of both natural and synthetic materials have shown promising bone healing results. Highly porous (~ 80%) polymer scaffolds with 100–500 µm interconnected pores consistently support new bone ingrowth when implanted in bone defects [209]. For instance, 3D-printed PCL scaffolds (often composite with hydroxyapatite) have been implanted in rat and sheep models; these implants not only integrated well, but also guided the formation of mineralized bone throughout their structure, especially when pore sizes were in the few-hundred-micron range. A recent study using a 3D printed PCL–HA scaffold with a Voronoi lattice (pore diameter ~ 300 µm) observed robust vascularized bone formation, with minimal fibrous tissue encapsulation [211]. Therefore, the consensus from many studies is that scaffold designs should include a distribution of pore sizes – e.g. an outer layer with finer features for cell attachment and an inner core with larger (~ 300 µm +) channels for vascularized bone ingrowth [208].

## Summary

This section further confirms that meso-scale topographical design is a key consideration in modern bone tissue engineering scaffolds. Incorporating 10–500 µm features in collagen hydrogels, composite polymer scaffolds, and other biomaterials has been shown to: (a) enhance osteoblast adhesion and homogeneous cell distribution, (b) promote osteogenic differentiation by providing an ECM-mimicking 3D context, and (c) dramatically improve in vivo bone regeneration through improved vascularization and tissue organization. These findings underscore that optimally engineered meso-architecture is crucial for creating functional bone grafts that closely emulate the structure and biology of native bone.

A majority of the studies referenced in this section focus on scaffold porosity and meso-scale spatial architectures designed to facilitate bone ingrowth and vascular regeneration. While such fully porous or hollow geometries may not directly apply to current solid-state dental implants, the underlying principles offer valuable direction for future design. Next-generation implants could incorporate meso-scale features—such as open pores, internal compartments, or surface-guided grooves—that mimic the 3D microenvironment shown to support osteogenic and vascular responses. Even without full penetration or interconnected porosity, strategically engineered meso-scale roughness or surface geometries may be sufficient to create cues and engage with cells and tissues. These findings provide critical insights into how surface design can move beyond traditional microroughness to orchestrate more comprehensive tissue-level interactions at the implant interface.

## Mechanical interlocking by meso-scale topography/roughness

Unlike nano- and micro-scale surface features that primarily influence cellular responses through biochemical cues—such as integrin binding and cytoskeletal signaling—meso-scale topography is expected to provide a distinct advantage: physical interlocking. These features, typically ranging from 10 to 500 µm in height or depth, create geometric complexities and undercuts and anchoring niches that support bone tissue ingrowth, thereby improving the implant’s primary stability, shear resistance, and overall anchoring capacity, as summarized in Fig. 6.

Experimental studies have demonstrated a direct link between meso-feature geometry and biomechanical performance by revealing significant correlation outcomes. High-temperature acid-etched titanium surfaces with progressively denser meso-spikes showed proportional increases in push-in strength, indicating stronger osseointegration through enhanced bone anchorage [167]. Likewise, zirconia implants with cactus-inspired spikes (approximately 40–60 µm in height) exhibited significantly greater removal torque, further supporting the mechanical contribution of meso-scale projections [201]. However, in both cases, the mechanical and biological effects are difficult to isolate completely, as the same features also enhance osteogenic cell behavior.

More direct evidence for mechanical interlocking comes from the study discussed in Sect. "Biological paradox of microroughness"., which performed controlled mechanical testing of titanium surfaces with and without sandblasting—an approach that introduces supramicro-scale roughness without altering surface microroughness and chemistry [117]. The results showed that sandblasting increased the interfacial shear strength between titanium and veneering resin by more than 3.5-fold. Moreover, surfaces that were pre-sandblasted and then acid-etched exhibited 1.5 times greater shear strength compared to acid-etched surfaces alone. These findings attribute the enhancement primarily to physical interlocking, not chemical bonding or contact area between titanium and materials.

Importantly, the study also revealed a strong positive correlation between mechanical retention of resin and the developed interfacial area ratio (Sdr)—a parameter that quantifies the percentage increase in surface area created by roughening. Higher Sdr values directly translated into improved interfacial adhesion. While the study focused on supramicro features, the principle scales upward: meso-scale structures naturally possess higher Sdr values than micro-scale textures, due to their larger vertical and lateral dimensions. Thus, the interlocking capacity of meso-textured surfaces is expected to be substantially greater. By extrapolation, meso-scale topographies with optimized Sdr could dramatically enhance mechanical fixation of implants, especially when combined with micro- and nano-scale features that foster biological integration. Although existing mechanical data largely come from resin–titanium interfaces, the same interfacial mechanics apply to bone–implant contact.

Thus, mechanical interlocking represents a crucial and often underappreciated advantage of meso-scale design. Beyond their biological impact, mechanical interlocking alone contributes structurally to implant fixation, especially under load-bearing conditions. The insights from the mechanical study and related research underscore the need to evaluate Sdr alongside traditional roughness metrics like Sa, and to develop implant surfaces that balance biological guidance with mechanical anchorage. Future studies are warranted to validate this hypothesis in bone-implant models and confirm the long-term mechanical benefits of high-Sdr meso-textured implants.

## Discussion

In vitro studies have established that surface topography across multiple scales influences cellular behavior in distinct and synergistic ways. Microroughness (~ 1–10 µm), smaller than cell size, provides biochemical cues and is known to enhance osteoblast differentiation while impairing proliferation and spreading. Nano-scale textures (< 1 µm) further modulate the effects of microroughness, fine-tuning cellular signaling and adhesion. Meanwhile, meso-scale structures (10–500 µm) were not initially expected to directly engage individual cells but instead to influence multicellular arrangement, extracellular matrix orientation, and tissue-level organization. Contrary to these expectations, meso-scale roughness has been shown to promote osteoblast differentiation, enhancing the effects of microroughness. Importantly, meso-scale topography does not impede proliferation despite its increased roughness; instead, it may promote it—effectively mitigating or even resolving the long-standing biological trade-off between osteoblast proliferation and differentiation. These distinct yet overlapping biological roles across scales are illustrated in Fig. 6.

Despite compelling experimental evidence, the application of meso-scale structuring in dental implants remains rare. This disconnect arises from multiple factors, including differences in material configuration, mechanical performance requirements, and manufacturing constraints between research models and clinical-grade implants. Most existing data are derived from scaffold systems or porous constructs, which differ substantially from the dense, solid-core design typical of dental implants. Introducing open meso-scale pores or channels into such implants could compromise mechanical integrity, create potential pathways for bacterial infiltration, and hinder retrievability in cases of failure or infection. In load-bearing contexts, particularly with narrow or tapered designs, large meso-cavities could undermine strength and fracture resistance. Furthermore, surface-connected pores may interfere with mechanical interlocking by interrupting continuity with the implant core. For these reasons, meso-scale features must be engineered as surface-level textures rather than bulk porosity and carefully balanced against structural demands.

The optimal strategy may therefore lie in hybridizing meso-scale roughness with conventional microroughening techniques. High-temperature acid etching and laser texturing have demonstrated the ability to generate robust, multi-scale surfaces while maintaining material chemistry and mechanical integrity. These subtractive methods can produce well-defined meso-scale topographies without introducing microcracks or structural compromise. Laser texturing, in particular, allows for precise, contact-free modification of solid-core implants—on both titanium and zirconia—enabling the creation of grooves, pits, and spiked arrays with tunable dimensions. These features can be superimposed onto existing microroughness and applied selectively to areas requiring enhanced bone anchorage. Data from in vitro and in vivo models support the clinical relevance of such multi-scale surfaces. For example, laser-generated meso-spikes on zirconia surfaces have demonstrated osteoblast proliferation and differentiation exceeding that of microrough titanium.

By contrast, additive manufacturing (AM) presents an alternative route for generating architecturally complex implant surfaces, but clinical translation faces substantial hurdles. In titanium, methods such as selective laser melting (SLM) and electron beam melting (EBM) can produce tailored meso-scale geometries. However, these often introduce residual stress, require complex post-processing, and exhibit surface and dimensional variability that compromises consistency in biological and clinical performance. In zirconia, post-printing sintering can cause shrinkage, collapse of fine pores, and decreased fracture toughness, making clinical application even more challenging. Additionally, AM-fabricated implants often exhibit inferior fatigue resistance compared to conventionally machined counterparts. At present, AM appears better suited for non-load-bearing scaffolds and preclinical constructs rather than functional dental implants.

Another critical barrier is the lack of standardized metrics for characterizing meso-scale surface topography. Traditional surface parameters—such as Sa, Ra, and Sdr—were developed for micro-scale textures and are often inadequate for capturing the spatial complexity of meso-scale features. Quantifying such topographies requires expanded scan areas, multi-resolution imaging, and the development of new analytical tools capable of resolving hierarchical surface morphologies. Many studies included in this review did not report quantitative metrics for meso-features, underscoring a major methodological limitation. For reproducibility, regulatory approval, and translational success, the field must adopt scale-appropriate standards and metrics for meso-topography characterization.

Ultimately, the goal is to leverage meso-scale topographies not to replace, but to complement microroughness—filling the gap between cell-level biochemical cues and macro-level mechanical design. The first biological priority is to overcome the osteoblastic trade-off that limits traditional microrough surfaces. Meso-topographies provide not only spatial guidance for multicellular organization but also functional advantages in cell proliferation, matrix deposition, and vascular infiltration. This integrated design approach mirrors the hierarchical architecture of native bone—offering a biomimetic solution to long-standing limitations in implant integration. Meso-scale engineering is more than a refinement; it represents a paradigm shift in the understanding and construction of the bone–implant interface. Thoughtfully applied, it holds the potential to accelerate osseointegration, shorten healing times, and enhance other clinical outcomes across broader patient populations. In the future, implant surfaces will not be defined by a single roughness value, but by their ability to harmonize biological, mechanical, and clinical performance across scales.

## Conclusion

This review has identified a critical and underutilized dimension in implant surface design: meso-scale topography and roughness. While micro-roughened surfaces have long been the clinical gold standard, their limitations—especially the biological trade-off between osteoblast proliferation and differentiation—have become increasingly evident. Despite substantial evidence supporting the biological value of micro- and nano-scale modifications, the meso-scale range (10–500 µm) remains largely unexplored in dental implants, representing a significant gap in both knowledge and technology.

Emerging data from biomedical research, along with recent advances in titanium and zirconia surface engineering, indicate that meso-scale features can complement existing designs by facilitating early osteoblast attachment, enhancing cellular proliferation, guiding matrix organization, promoting vascular infiltration, and improving mechanical interlocking with host bone. Unlike micro-scale textures that primarily regulate intracellular signaling pathways, meso-topographies orchestrate multicellular organization and tissue-level integration. They offer expanded spatial environments and physical cues that can mitigate the longstanding proliferation–differentiation dichotomy.

The current underuse of meso-scale topography stems from both conceptual oversight and technical limitations. A deeper understanding of its biological impact is needed, along with improvements in manufacturing methods capable of integrating complex meso-scale geometries into solid-core implants without compromising their mechanical integrity. Translation into clinical devices will require innovations in processing techniques—such as high-precision laser texturing, high-temperature acid etching, or additive manufacturing—and a shift in design philosophy that embraces true multiscale integration.

Next-generation implant surfaces will evolve from single-scale optimization to hierarchically structured designs that strategically combine micro- and meso-scale features. This integrative approach has the potential to synergistically enhance biological responses, improve mechanical fixation, accelerate early healing, and extend long-term success. Far from being a minor refinement, meso-scale engineering represents a paradigm shift—one that redefines the biological–mechanical interface and transforms the foundation of osseointegration by bridging the missing middle.

## Data Availability

No datasets were generated or analysed during the current study.
